# Effects of resveratrol in an animal model of osteoporosis: a meta-analysis of preclinical evidence

**DOI:** 10.3389/fnut.2023.1234756

**Published:** 2023-07-27

**Authors:** Jinlong Zhao, Guanghui Zhou, Junzheng Yang, Jianke Pan, Bangxin Sha, Minghui Luo, Weiyi Yang, Jun Liu, Lingfeng Zeng

**Affiliations:** ^1^The Second Clinical College of Guangzhou University of Chinese Medicine, Guangzhou, China; ^2^Guangdong Provincial Hospital of Chinese Medicine, Guangzhou, China; ^3^The Research Team on Bone and Joint Degeneration and Injury of Guangdong Provincial Academy of Chinese Medical Sciences, Guangzhou, China; ^4^The Fifth Clinical College of Guangzhou University of Chinese Medicine, Guangzhou, China; ^5^Guangdong Second Chinese Medicine Hospital (Guangdong Province Enginering Technology Research Institute of Traditional Chinese Medicine), Guangzhou, China

**Keywords:** resveratrol, plant-based natural products, osteoporosis, bone mineral density, meta-analysis, evidence-based medicine

## Abstract

**Background:**

Resveratrol is a natural polyphenol compound that is widely present in herbal medicines such as *Reynoutria japonica Houtt.*, *Veratrum nigrum L.*, and Catsiatora Linn and is used in traditional Chinese medicine to treat metabolic bone deseases. Animal experiments have shown that resveratrol may have a strong treatment effect against osteoporosis (OP). The purpose of this study was to explore the efficacy of resveratrol in treating OP animal models based on preclinical research data.

**Methods:**

This study was completed according to the Preferred Reporting Items for Systematic Reviews and Meta-Analyses (PRISMA) guidelines. We searched the PubMed, Embase, Cochrane Library, and China National Knowledge Infrastructure (CNKI) databases from inception to May 8, 2023, to identify animal experiments on the treatment of OP with resveratrol. The effect sizes of bone mineral density (BMD), parameters of micro-CT, serum calcium, phosphorus, alkaline phosphatase (ALP) and osteocalcin were expressed as the mean differences (MDs) and 95% confidence intervals (CIs). RevMan 5.4 software was used for data analysis.

**Results:**

This meta-analysis included a total of 15 animal experiments, including 438 OP rats. The meta-analysis results showed that compared with the control group, resveratrol (<10, 10–25, 40–50, ≥ 60 mg/kg/day) significantly increased femoral and lumbar bone mineral density (BMD) in OP rats (*p* < 0.05). Resveratrol (<10 mg/kg/day) significantly increased the BMD of the total body (MD = 0.01, 95% CI: 0.01 to 0.01, *p* < 0.001). In terms of improving the parameters related to micro-CT, resveratrol (40–50 mg/kg/day) can increase trabecular thickness and trabecular number and reduce trabecular spacing (*p* < 0.05). Compared with the control group, resveratrol can reduce the concentration of calcium and phosphorus in serum but has no significant effect on serum ALP and osteocalcin (*p* > 0.05). The results of subgroup analysis showed that resveratrol increased the whole-body BMD of SD rats (*p* = 0.002) but did not improve the whole-body BMD of 3-month-old rats (*p* = 0.17).

**Conclusion:**

Resveratrol can increase BMD in OP rat models, and its mechanism of action may be related to improving bone microstructure and regulating calcium and phosphorus metabolism. The clinical efficacy of resveratrol in the treatment of OP deserves further research.

## 1. Introduction

Osteoporosis (OP) is a systemic bone disease characterized by low bone mass, damage to the microstructure of bone tissue, and increased bone fragility (1). The increased risk of bone fragility and fracture caused by OP poses a heavy economic burden to society and patients (2, 3). The aetiology of OP is complex and diverse, including the interactions between endocrine, nutritional, genetic, physiological, and immune factors (4). Among them, postmenopausal osteoporosis (PMOP) is considered strongly correlated with oestrogen deficiency (5). The increase in bone resorption and the decrease in bone formation lead to an imbalance in bone homeostasis (1, 6), which is closely related to the occurrence of OP. An epidemiological study has shown that the prevalence of OP in people over 50 years old in Europe and America is 4–6%, while in Asian populations, it is over 15% (7). According to the diagnostic criteria of the World Health Organization (WHO), the latest epidemiological research results show that the global prevalence of OP is as high as 19.7% (8, 9). The OP prevalence rates in different countries (4.1% in Netherlands to 52.0% in Türkiye) and continents (8.0% in Oceania to 26.9% in Africa) vary greatly (8, 9). As the population continues to age, OP is recognized as a major public health issue (7). At present, the treatment of OP mainly includes bisphosphonates, parathyroid drugs, or oestrogen replacement therapy (10, 11), all of which have inevitable adverse reactions. Therefore, researching and developing more alternative drugs with fewer side effects and better therapeutic effects is an important topic for OP treatment.

Botanical or traditional medicine have always been breakthrough points in new drug development, mainly due to their higher potential for drug conversion and lower incidence of adverse reactions. Traditional Chinese medicine is also commonly used for the treatment of OP, and its pharmacological mechanism usually has the characteristics of “multiple components, multiple targets, and multiple pathways.” Resveratrol is a natural polyphenol compound with a structure similar to oestrogen diethylstilbestrol, which is widely present in herbs such as *Reynoutria japonica Houtt.*, *Veratrum nigrum L.*, and *Catsiatora Linn* (12, 13). Research has shown that resveratrol competitively binds to oestrogen receptors *in vitro*, similar to phytoestrogens, and exerts anti-OP effects (14). Another study showed that resveratrol can affect the metabolism of bone cells and has the ability to regulate bone turnover (15). It is a natural antioxidant that can effectively prevent bone loss caused by oxidative stress in the body. Previous clinical studies have suggested that resveratrol can reduce bone loss and fracture risk in postmenopausal women or diabetes patients (16, 17). However, there is currently a lack of advanced evidence for the use of resveratrol in the treatment of OP; therefore, there is a lack of clarity regarding the application value of resveratrol.

The pre-clinical studies conclusions of animal experiments can provide key information for clinical practice and enhance the understanding of disease mechanisms among clinical and scientific researchers. At present, the clinical evidence for the treatment of OP with resveratrol is very limited, and thus, there is little information regarding the potential medicinal value of resveratrol in OP treatment. However, in the experimental field, studies have examined resveratrol treatment of OP animal models. This systematic review and meta-analysis aimed to evaluate the efficacy of resveratrol in treating OP animal models in order to provide evidence for future research on the anti-OP clinical efficacy of resveratrol.

## 2. Materials and methods

The implementation of this study strictly followed the Preferred Reporting Items for Systematic Reviews and Meta-Analyses (PRISMA) guidelines (18). The data source for this meta-analysis is publicly published papers, which means that ethical review was not needed.

### 2.1. Eligibility criteria

The inclusion criteria for this meta-analysis were as follows: (1) the study design was a controlled experiment, which means that the study protocol included both an experimental group and a control group, (2) the research object was a female rat model; the species of rats were Albino rats, SD rats, or Wistar rats; the age of rats did not exceed 6 months, and the modelling method was ovariectomy (OVX), (3) the intervention for the experimental group was resveratrol, but the dosage of resveratrol was not limited, (4) comparison: the intervention measures for the control group can be blank control (tap water or normal saline) or other drug treatments, and (5) outcome index: bone mineral density (BMD) (g/cm^2^) is the primary outcome measure, and secondary outcomes included trabecular thickness (Tb. Th), trabecular number (Tb. N), trabecular spacing (Tb. SP), serum calcium (mmol/l), serum phosphorus (mmol/l), serum alkaline phosphatase (ALP) (U/l), and serum osteocalcin (nmol/l); furthermore, all outcome indicators must clearly report the results of the measurement data, and the data reporting format must be mean ± standard deviation. There were no restrictions regarding publication language.

### 2.2. Exclusion criteria

The exclusion criteria were as follows: (1) review, meeting abstract, and case report, (2) incomplete experimental data, and (3) *in vitro* studies or clinical studies.

### 2.3. Search strategies

We searched the following four databases to obtain animal experimental studies on the treatment of OP with resveratrol: PubMed, Embase, The Cochrane Library, and China National Knowledge Infrastructure (CNKI). The search was performed from database inception to May 8, 2023. The search strategy included a combination of MeSH terms and free words, and the strategy was adjusted based on the characteristics of each database. The keywords related to resveratrol included “Resveratrol” OR “trans-Resveratrol” OR “3 5 4 trihydroxystilbene” OR “cis-Resveratrol” OR “3 4 5 stilbenetriol” OR “trans-Resveratrol-3-O-sulfate” OR “trans-Resveratrol-3-O-sulfate” OR “SRT-501” OR “trans-Resveratrol” OR “SRT501” OR “SRT-501” OR “cis-Resveratrol” OR “Resveratrol-3-sulfate” OR “3 4 5 trihydroxystilbene” OR “Resveratrol-3-sulfate.” The keywords for OP include: “Osteoporosis” OR “OP” OR “Osteoporoses” OR “bone loss” OR “bone density” OR “bone mineral density” OR “bone mass density.”

### 2.4. Data extraction

Two researchers independently conducted literature screening and data extraction and cross-checked the results. Disagreements were resolved by discussion or by consulting a third researcher. The following data were extracted: (1) basic information of the included study: author, title, year of publication, animal species, weight, age, and sample size, (2) specific details of intervention measures, including medication dosage and duration, (3) the various information elements of bias risk assessment, and (4) outcome indicators and outcome measurement data.

### 2.5. Quality evaluation of the included studies

We used the risk of bias tool for animal studies provided by the Systematic Review Center for Laboratory Animal Experience (SYRCLE) to conduct a literature quality evaluation of the included studies (19, 20). This evaluation tool has a total of 9 items, including random group allocation, groups similar at baseline, blinded group allocation, random housing, blinded interventions, random outcome assessment, blinded outcome assessment, reporting of drop-outs, and other biases. Each item can be judged as having low bias risk, high bias risk, and unclear bias risk (19, 20).

### 2.6. Statistical analysis

RevMan 5.4 software was used for data analysis. The outcome measures included in this study were all continuous variables, so all combined effects are expressed as the mean difference (MD) and 95% confidence interval (CI). This meta-analysis used the random-effects model for pooled data analysis. To clarify the anti-OP effect of resveratrol at different doses, we divided the drug doses of resveratrol into four groups: >10, 10–25, 40–50, and ≥ 60 mg/kg/day. In each included study, if there were 2 or more sets of satisfactory measurement data within the same dose range (the same study), the group with the lowest dose was selected for meta-analysis. Considering that differences in race and age of rats may affect the reliability of the conclusion, we conducted subgroup analyses based on those two factors. In particular, the resveratrol group used in the subgroup analysis was the lowest-dose group in each included study. We also constructed funnel plots for each outcome indicator to evaluate potential publication bias.

## 3. Results

### 3.1. Literature screening results

**Figure 1:**
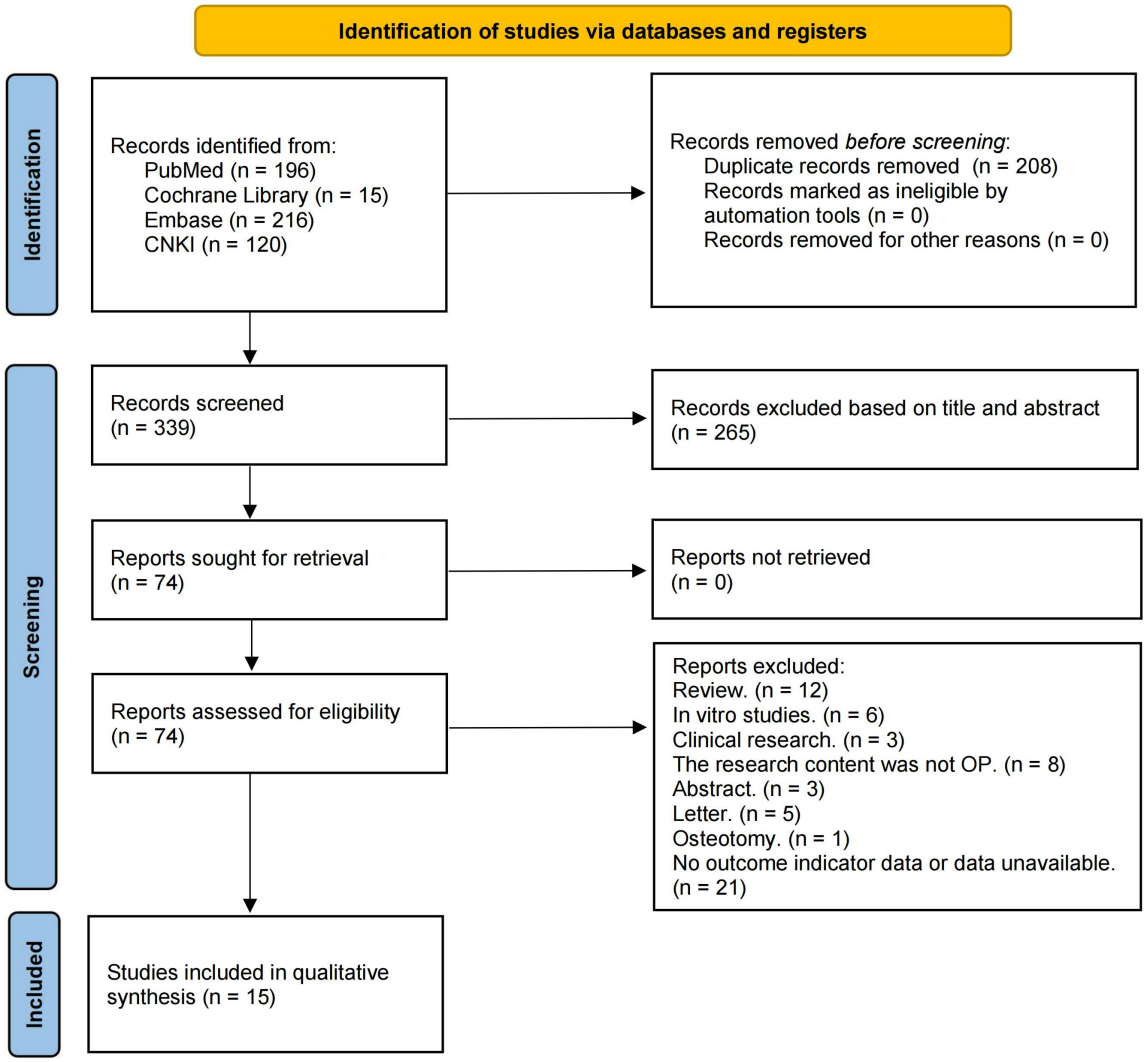
Summary of the process for identifying candidate studies.

After removing duplicate literature, we initially obtained 339 articles. In the initial screening, we excluded literature that clearly did not meet the inclusion criteria based on the information provided by the title and abstract. After applying the inclusion and exclusion criteria and screening full texts, a total of 15 studies on the treatment of OP animal models with resveratrol that met the requirements of this meta-analysis were ultimately included (21–35). The search process and details are shown in Figure 1.

### 3.2. Characteristics of the 15 included studies

This meta-analysis included 15 experimental studies on the treatment of OP rats with resveratrol. A total of 438 rats were included in this study, including 295 in the resveratrol group and 143 in the control group. There are three types of rat strains, namely, Albino rats, SD rats, and Wistar rats. The modelling method for OP is OVX. The dosage of resveratrol varies greatly, with a minimum dosage of 625 μg/kg/day and a maximum dosage of 500 mg/kg/day. The course of medication is between 4 and 24 weeks. The specific details and characteristics of each included study are shown in Table 1.

**Table 1:** Characteristics of the 15 included studies.

Study	Model (method)	Species	Age	Weight (g)	Intervention		Duration	Sample size	
					Resveratrol	Control		Resveratrol	Control
Elseweidy et al. (21)	OVX	Albino rats	3 months	200–220	80 mg/kg/day	Tap water	8 weeks	10	10
Feng et al. (22)	OVX	SD rats	3 months	280–350	5/25/45 mg/kg/day	Tap water	8 weeks	8/8/8	8
Feng et al. (23)	OVX	SD rats	3 months	220 ± 19.27	40 mg/kg/day	Sesame oil	10 weeks	10	10
Guo et al. (24)	OVX	Wistar rats	8 weeks	180–200	500 mg/kg/day	Tap water	60 days	10	10
Khera et al. (25)	OVX	SD rats	3 months	NR	625 μg/kg/day	Tap water	4 weeks	6	6
Li et al. (26)	OVX	SD rats	6 months	250 ± 20	10/20/40 mg/kg/day	Tap water	12 weeks	6/6/6	6
Lin et al. (27)	OVX	SD rats	3 months	254.91 ± 18.01	5/15/45 mg/kg/day	Tap water	90 days	8/8/8	8
Liu et al. (28)	OVX	Wistar rats	NR	220–250	0.7 mg/kg/day	Tap water	12 weeks	11	11
Wang et al. (29)	OVX	SD rats	NR	NR	10/20/40 mg/kg/day	Tap water	8 weeks	8/8/8	8
You et al. (30)	OVX	Wistar rats	6 weeks	NR	8.4 mg/kg/day	Tap water	8 weeks	10	10
Zhang et al. (31)	OVX	SD rats	6 months	220 ± 10	5/15/45 mg/kg/day	Tap water	12 weeks	12/12/12	12
Zhang et al. (32)	OVX	SD rats	6 months	NR	50/100/200 mg/kg/day	Carboxymethyl cellulose	12 weeks	8/8/8	8
Zhang et al. (33)	OVX	SD rats	6 weeks	241.06 ± 32.81	40 mg/kg/day	Tap water	8 weeks	10	10
Zhao et al. (34)	OVX	Wistar rats	3–4 months	200–220	20/40/80 mg/kg/day	Tap water	12 weeks	10/10/10	10
Zhou et al. (35)	OVX	SD rats	6 months	360 ± 10	60/80/100 mg/kg/day	Tap water	24 weeks	16/16/16	16

OVX, ovariectomy; SD, Sprague–Dawley; NR, not reported.

### 3.3. Literature quality evaluation

**Figure 2:**
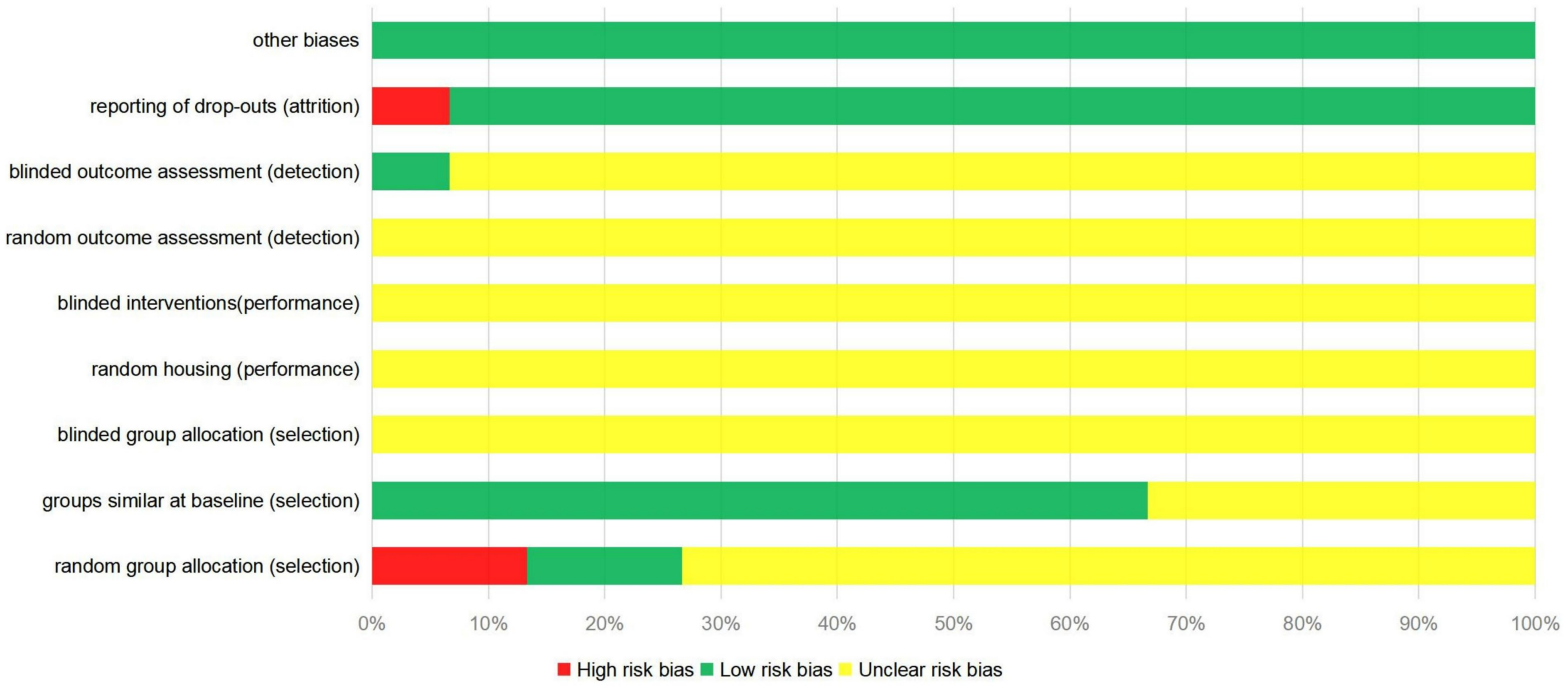
Risk of bias of the 15 included studies.

Most of the 15 studies included in this meta-analysis were evaluated for unclear risk bias. Only 2 studies used the random number table method (30, 33); 2 studies did not use random assignment (21, 27); and the remaining studies did not provide sufficient information to determine whether the experimental animals were randomly assigned. One study used a blinding method for the evaluators of results (22). One study did not provide a detailed explanation of missing data (29), which may lead to potential data reporting bias. The quality evaluation results of the literature included in the study are shown in Figure 2.

### 3.4. Results of meta-analysis

#### 3.4.1. Primary outcomes

##### 3.4.1.1. BMD of the total body

**Figure 3:**
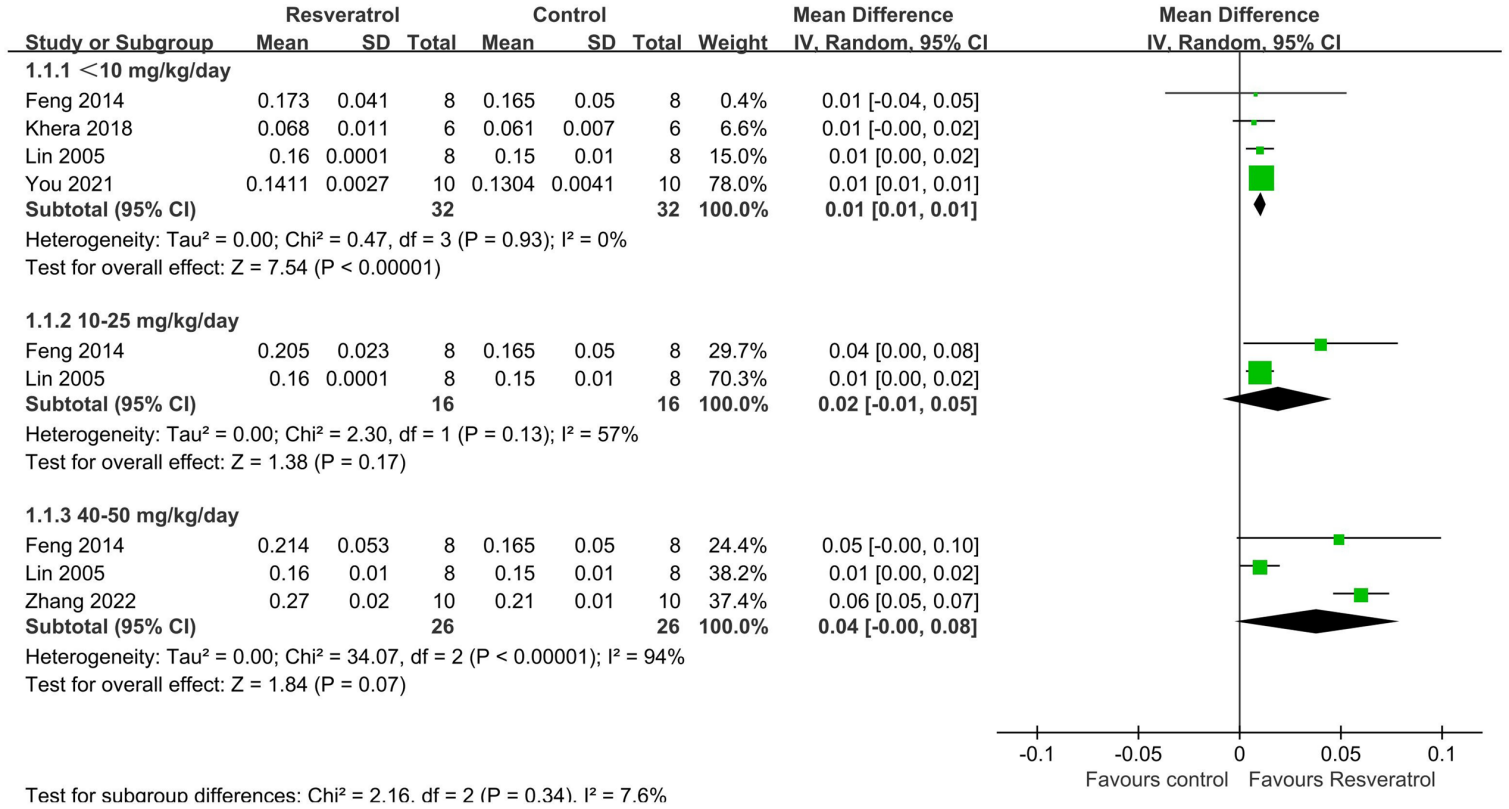
Forest plot of the total body BMD.

A total of 5 studies (22, 25, 27, 30, 33) reported total-body BMD (Figure 3). The meta-analysis results showed that compared with the control condition, <10 mg/kg/day resveratrol significantly increased the total-body BMD of the OP rat model (MD = 0.01, 95% CI: 0.01 to 0.01; *p* < 0.001), and there was no heterogeneity among the studies in this subgroup (*I*^2^ = 0%). However, resveratrol doses of 10–25 mg/kg/day and 40–50 g/kg/day showed no significant difference in total-body BMD compared with the control condition (*p* > 0.05).

##### 3.4.1.2. BMD of the femur

**Figure 4:**
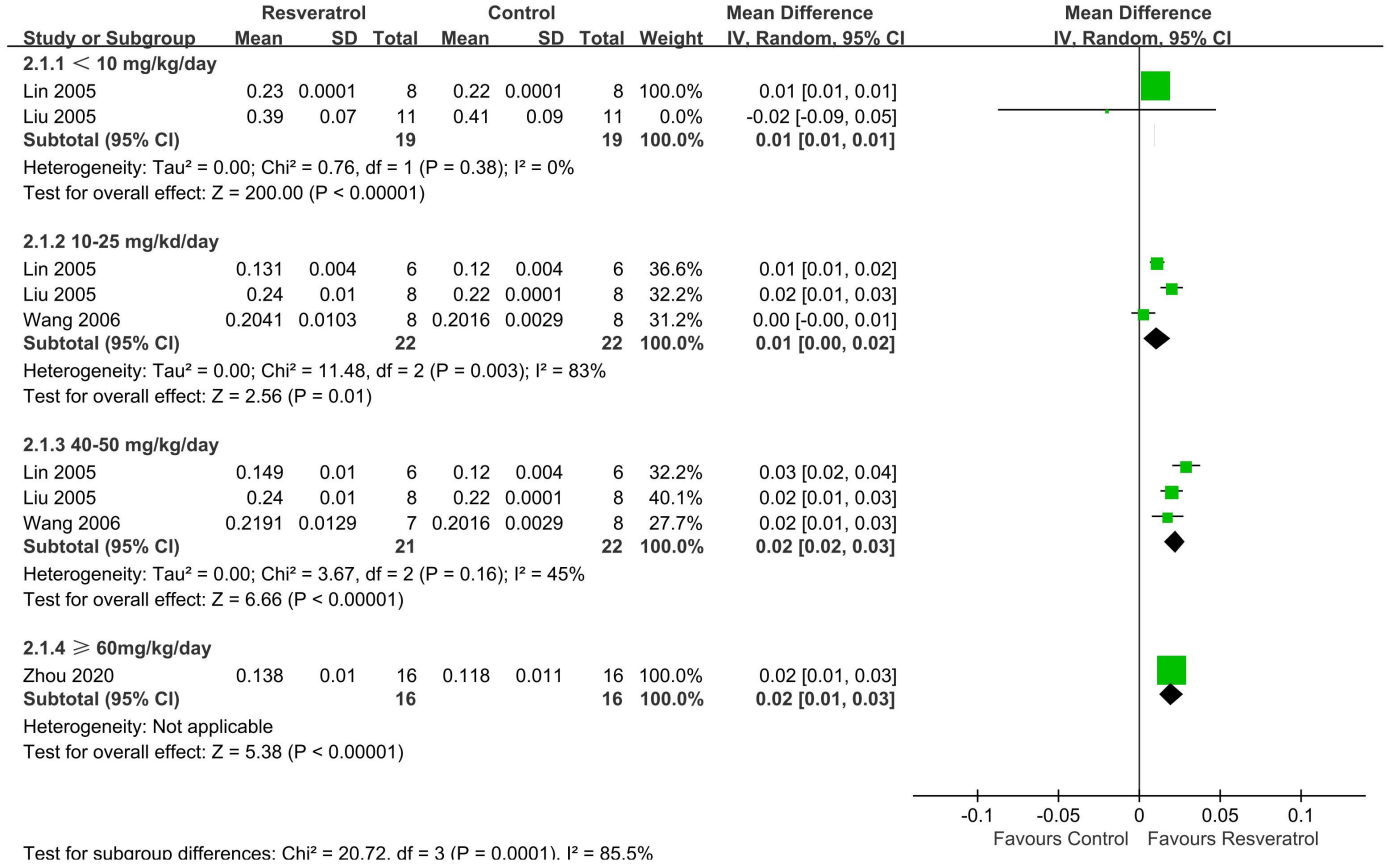
Forest plot of femur BMD.

A total of 5 studies (26–29, 35) reported FBMD (Figure 4). The meta-analysis results showed that compared with the control group, four doses of resveratrol (<10, 10–25, 40–50, ≥ 60 mg/kg/day) all increased FBMD in OP rats, with MDs (95% CIs) of 0.01 (0.01, 0.01), 0.01 (0.00, 0.02), 0.02 (0.02, 0.03), and 0.02 (0.01, 0.03), respectively.

##### 3.4.1.3. BMD of the lumbar vertebrae

**Figure 5:**
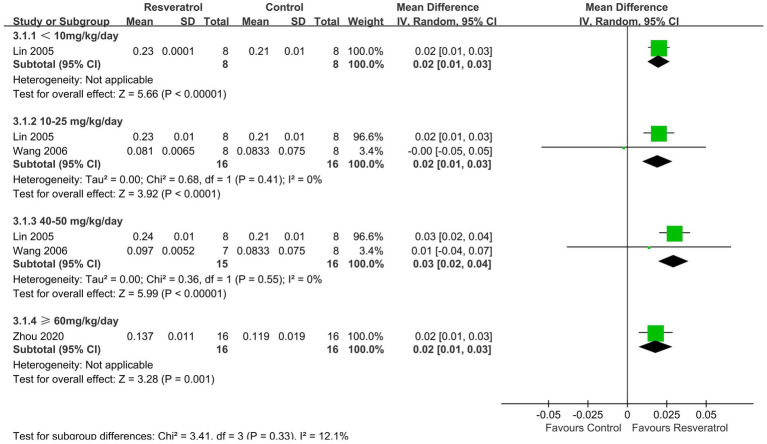
Forest plot of lumbar vertebrae BMD.

Three studies (27, 29, 35) reported LBMD (Figure 5). The meta-analysis results showed that resveratrol <10 (MD = 0.02, 95% CI: 0.01 to 0.03), 10–25 (MD = 0.02, 95% CI: 0.01 to 0.03), 40–50 (MD = 0.03, 95% CI: 0.02 to 0.04), and ≥ 60 (MD = 0.02, 95% CI: 0.01 to 0.03) mg/kg/day significantly increased LBMD compared to the control group (*p* < 0.05).

#### 3.4.2. Secondary outcomes

##### 3.4.2.1. Parameters of micro-CT

**Figure 6:**
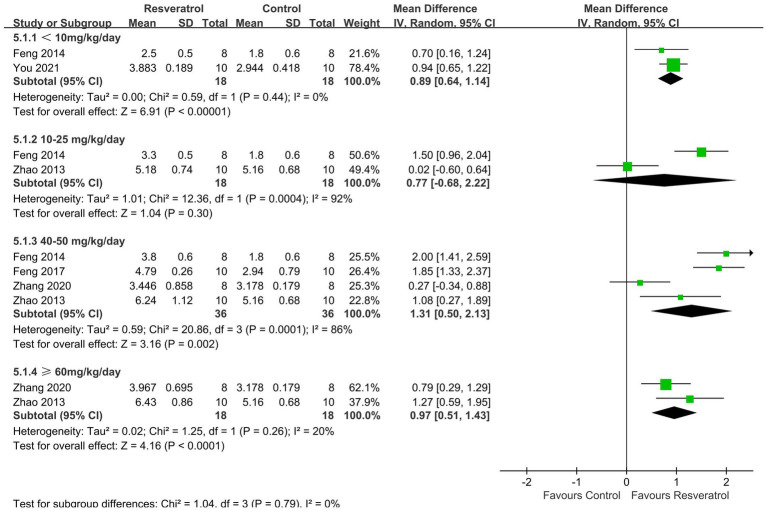
Forest plot of trabecular number.

This meta-analysis analysed three parameters related to micro-CT, namely, Tb. Th (Supplementary material 1), Tb. N (Figure 6), and Tb. Sp (Supplementary material 2). The meta-analysis results showed that resveratrol (40–50 mg/kg/day) significantly increased Tb. Th (MD = 0.01, 95% CI: 0.01 to 0.01) in the OP rat model. Compared with the control group, resveratrol (<10, 40–50, ≥ 60 mg/kg/day) increased Tb. N (*p* < 0.05). Resveratrol (10–25, 40–50, ≥60 mg/kg/day) was more effective in reducing Tb. Sp compared to the control group, and the differences were statistically significant (*p* < 0.05).

##### 3.4.2.2. Serum calcium

**Figure 7:**
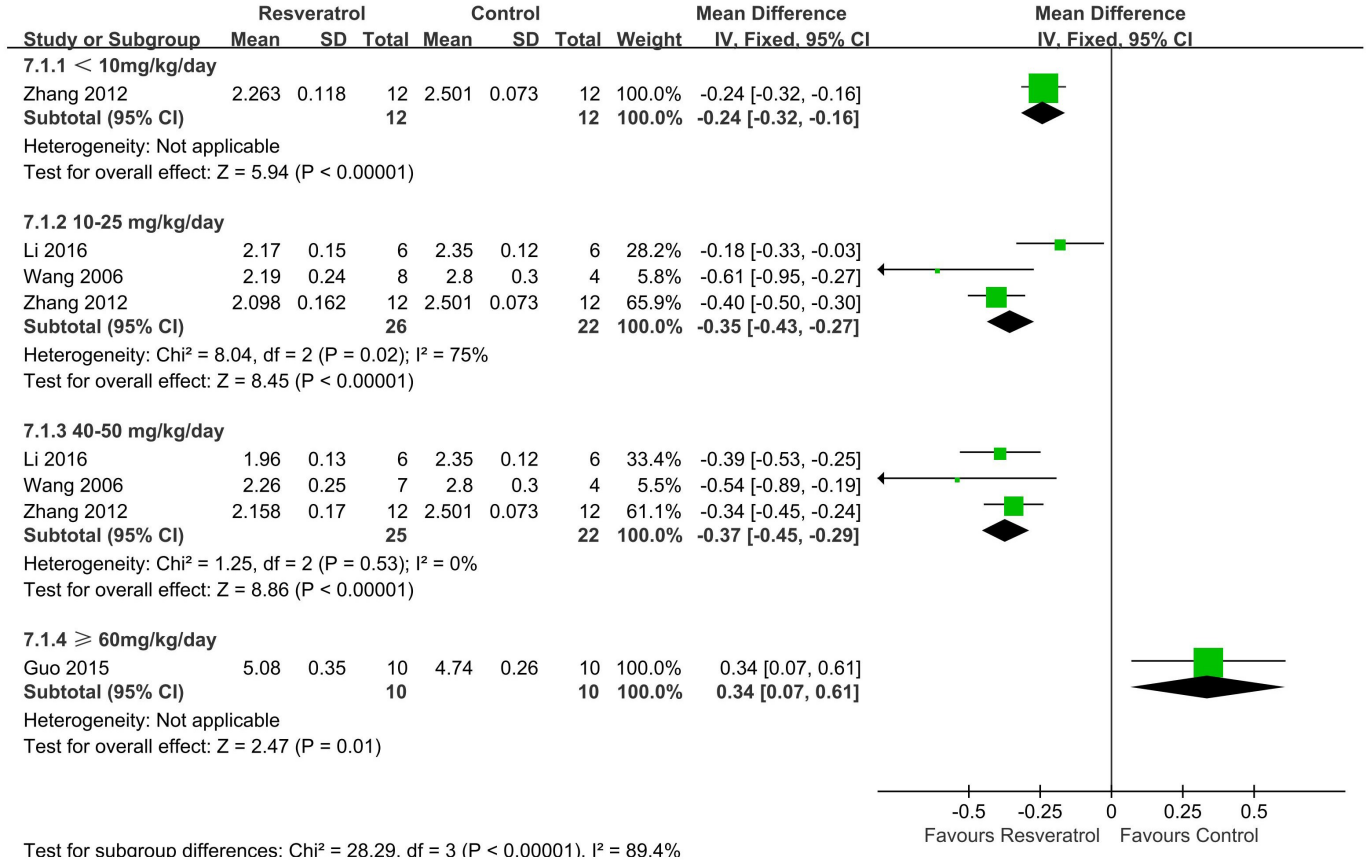
Forest plot of serum calcium.

Four studies (24, 26, 29, 31) reported changes in serum calcium concentration (Figure 7). The meta-analysis results showed that resveratrol at concentrations of <10 (MD = -0.24, 95% CI: −0.32 to-0.16), 10–25 (MD = -0.35, 95% CI: −0.43 to-0.27), and 40–50 (MD = -0.37, 95% CI: −0.45 to-0.29) mg/kg/day significantly reduced serum calcium concentration compared to the control group (*p* < 0.001).

##### 3.4.2.3. Serum phosphorus

Similarly, four studies (24, 26, 29, 31) reported changes in serum phosphorus concentration (Supplementary material 3). The meta-analysis results showed that resveratrol was more effective in reducing serum phosphorus concentration compared to the control group, and the differences were statistically significant (*p* < 0.05).

##### 3.4.2.4. Serum ALP

**Figure 8:**
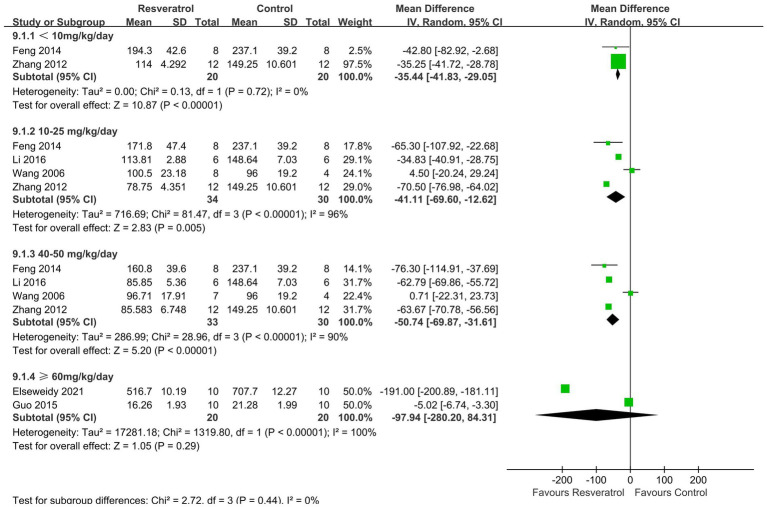
Forest plot of serum alkaline phosphatase.

A total of 6 studies (21, 22, 24, 26, 29, 31) reported serum ALP levels (Figure 8). The meta-analysis results showed that there was no statistically significant difference in the effect of resveratrol and the control group on serum ALP (*p* > 0.05).

##### 3.4.2.5. Serum osteocalcin

A total of 4 studies (22, 24, 30, 33) reported changes in serum osteocalcin levels (Supplementary material 4). The meta-analysis results showed that there was no significant difference in the effect of resveratrol on serum osteocalcin compared to the control group (*p* > 0.05).

#### 3.4.3. Subgroup analysis of BMD of the total body

##### 3.4.3.1. Resveratrol in SD rats

**Figure 9:**
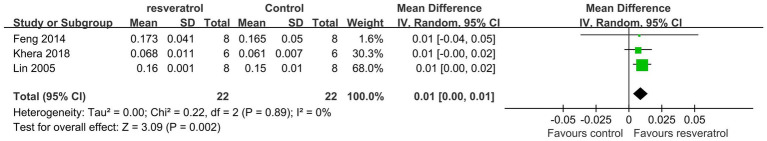
Subgroup analysis of total-body BMD in SD rats.

A total of 3 studies were included in the subgroup analysis (22, 25, 27). Meta-analysis results showed that compared with the control condition, resveratrol treatment resulted in a statistically significant increase in total-body BMD (MD = 0.01, 95% CI: 0.00 to 0.01; *p* = 0.002) (Figure 9).

##### 3.4.3.2. Resveratrol in 3-month-old rats

**Figure 10:**
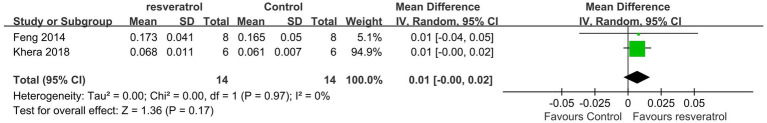
Subgroup analysis of total-body BMD in 3 months aged rats.

A total of 2 studies were included in the subgroup analysis (22, 25). Meta-analysis results showed that compared with the control treatment, resveratrol treatment had no statistically significant effect on improving total-body BMD (*p* = 0.17) (Figure 10).

### 3.5. Publication bias

We plotted corresponding funnel plots for all outcome indicators to evaluate publication bias. The funnel plot results show that the funnel plots of BMD of the total body, Tb. Th, serum phosphorus, and serum osteocalcin are asymmetric, indicating that there may be publication bias in these outcome indicators. The funnel plot of all outcome indicators is shown in Supplementary material 5.

## 4. Discussion

OP is known as the silent killer, and osteoporotic fractures are a serious complication of OP, which means that the prevention and treatment of OP are important aspects to which public health needs to pay attention. Ethnic medicine or botanical medicine has always been the focus of drug conversion. In recent years, the therapeutic effect of resveratrol on OP has received considerable attention, but research on its anti-OP efficacy or mechanism is mostly limited to animal or cell experiments, which seriously limits the progress of resveratrol in clinical application. To further clarify the anti-OP efficacy of resveratrol, this study summarizes preclinical evidence to provide support to proceed with clinical trials. This meta-analysis found that resveratrol can significantly increase FBMD and LBMD in OP rats, and this conclusion remained consistent at concentrations <10, 10–25, 40–50, and ≥ 60 mg/kg/day. In the improvement of BMD of the total body, resveratrol (<10 mg/kg/day) showed better efficacy than the control group. In terms of improving the parameters related to micro-CT, resveratrol can increase Tb. Th and Tb. N and reduce Tb.Sp. The concentration of resveratrol at 40–50 mg/kg/day can all improve these three bone microstructure indicators. In addition, resveratrol can reduce the concentration of calcium and phosphorus in serum but has no significant effect on serum ALP and osteocalcin, which was also verified in this meta-analysis. Based on preclinical animal research data, we found that resveratrol may have enormous clinical application potential in the treatment of OP, which means that resveratrol may become a candidate drug for OP treatment, but this still needs to be verified through large-scale clinical studies in the future.

Resveratrol has the characteristics of multiple targets, low cost, and low toxicity (36), and its therapeutic effect in OP is receiving increasing attention. The dynamic balance between osteoblasts and osteoclasts has always been considered the core content of OP research. An experimental study found that resveratrol can activate the osteogenic transcription factor CBFA-1 (37) and enhance the transcription of bone-specific type I collagen in a CBFA-1-dependent manner, stimulate the proliferation and differentiation of osteoblasts, and activate Sirt-1 to transform osteoblasts into osteoblasts. Research shows that resveratrol can upregulate the expression level of Sirt-1 and then upregulate the expression of FoxO1 protein to inhibit the differentiation of osteoclasts (38). The occurrence of oxidative stress can cause damage to bone cells and osteoblasts (39, 40) and lead to bone resorption activity exceeding bone formation. Resveratrol is a natural antioxidant and can effectively prevent bone loss caused by oxidative stress in the body (15), which may be the potential mechanism of its anti-OP effect. In addition, resveratrol can bind to oestrogen receptors and exert oestrogenic effects (32), thus compensating for bone loss caused by oestrogen deficiency. In addition, this meta-analysis showed that resveratrol achieved better efficacy in improving biochemical markers. Serum biochemical indicators reflect the essence of bone metabolism and the direct reflection of bone formation and bone resorption. This meta-analysis found that resveratrol has a better effect than the control treatment in reducing serum calcium concentration, which may be because resveratrol inhibits oxidative stress and reduces bone loss, thereby reducing the content of calcium entering the serum. Oxidative stress may lead to oxidative damage to bone cells and osteoblasts in the bone microenvironment, leading to imbalanced bone remodelling. The antioxidant effect of resveratrol can maintain bone homeostasis, thus stabilizing bone microstructure. Based on the undeniable regulatory role of resveratrol in bone metabolism, its clinical application in OP deserves in-depth attention.

## 5. Limitations

This study has limitations that should be considered when interpreting the results. First, the animal models included in the study may exhibit significant differences in factors such as species of rats, drug dosage, and sample size, which may lead to heterogeneity in the experiment and compromise the reliability of the conclusions of this study. Second, the included animal experiment reports focus on the construction of animal models and outcome evaluation, but the report on experimental design, implementation, and measurement methods is relatively brief, which may lead to poor methodological quality in literature reports, difficulty in estimating potential bias risks, and reduced data credibility. Third, there may be differences in the BMD measurement tools and serum markers used in all 15 included studies, which may lead to measurement errors between studies. Given the limitations of animal experimental design, future clinical studies targeting the treatment of OP with resveratrol should avoid these situations, which would be beneficial for improving the reliability of evidence-based data on this research topic.

## 6. Conclusion

This study found that resveratrol can increase BMD in OP rat models, and its mechanism of action may be closely related to improving bone microstructure and regulating calcium and phosphorus metabolism. Given that this study focuses on an OP rat model, the efficacy of resveratrol in treating OP still needs to be further validated through clinical studies in the future.

## Data availability statement

The original contributions presented in the study are included in the article/Supplementary material, further inquiries can be directed to the corresponding authors.

## Author contributions

JZ: conceptualization, validation, data curation, writing – original draft, writing – review and editing, visualization, supervision, and project administration. GZ and JY: writing – original draft, and writing – review and editing. JP: investigation, data curation, and formal analysis. BS and ML: investigation and data curation. WY: conceptualization, methodology, software, validation, formal analysis, and data curation. JL: conceptualization, supervision, validation, and funding acquisition. LZ: conceptualization, methodology, software, validation, formal analysis, data curation, writing – original draft, writing – review and editing, visualization, supervision, and funding acquisition. JZ and GZ: contributed equally to this work. All authors contributed to the article and approved the submitted version.

## Funding

This work was supported by the National Natural Science Foundation of China (No. 82004383, 82004386), Guangdong Basic and Applied Basic Research Foundation (No. 2022A1515220131, 2022A1515010385, 2022A1515011700), the National key research and development program (2021YFC1712804), Research Fund for Bajian Talents of Guangdong Provincial Hospital of Chinese Medicine (No. BJ2022KY01), Project of Philosophy and Social Science Planning of Guangzhou (No. 2022GZQN42), Foreign Teacher Programs of Guangdong Provincial Department of Science and Technology (YKZZ [2022] No. 232) and Administration of Traditional Chinese Medicine of Guangdong Province (No. 20231109, 20225025).

## Conflict of interest

The authors declare that the research was conducted in the absence of any commercial or financial relationships that could be construed as a potential conflict of interest.

## Publisher’s note

All claims expressed in this article are solely those of the authors and do not necessarily represent those of their affiliated organizations, or those of the publisher, the editors and the reviewers. Any product that may be evaluated in this article, or claim that may be made by its manufacturer, is not guaranteed or endorsed by the publisher.

## Abbreviations

OP: osteoporosis
CNKI: China National Knowledge Infrastructure
MD: mean difference
CI: confidence interval
BMD: bone mineral density
PMOP: postmenopausal osteoporosis
PRISMA: Preferred Reporting Items for Systematic Reviews and Meta analyses
NR: not reported
Tb. Th: trabecular thickness
Tb. N: trabecular number
Tb. SP: trabecular spacing
ALP: alkaline phosphatase
SYRCLE: Systematic Review Center for Laboratory Animal Experience
OVX: ovariectomy
SD: Sprague–Dawley
