# Supplementary material for: Effects of resveratrol in an animal model of osteoporosis: a meta-analysis of preclinical evidence
Source: Front Nutr. 2023 Jul 27;10:1234756. doi: 10.3389/fnut.2023.1234756 (PMC10414791; doi:10.3389/fnut.2023.1234756)
Supplement: Supplementary file 1 [file Data_Sheet_1.PDF]

Supplementary material 1 Forest plot of trabecular thickness.

	Resveratrol			Control				Mean Difference
**Study or Subgroup**	**Mean**	**SD**	**Total**	**Mean**	**SD**	**Total**	**Weight**	**IV, Random, 95% CI**
**4.1.1 < 10mg/kg/day**								
You 2021	0.187	0.006	10	0.184	0.007	10	100.0%	0.00 [-0.00, 0.01]
**Subtotal (95% CI)**			**10**			**10**	**100.0%**	**0.00 [-0.00, 0.01]**
Heterogeneity: Not applicable								
Test for overall effect: Z = 1.03 (P = 0.30)								
**4.1.2 10-25 mg/kg/day**								
Zhao 2013	0.04296	0.00312	10	0.04329	0.0021	10	100.0%	-0.00 [-0.00, 0.00]
**Subtotal (95% CI)**			**10**			**10**	**100.0%**	**-0.00 [-0.00, 0.00]**
Heterogeneity: Not applicable								
Test for overall effect: Z = 0.28 (P = 0.78)								
**4.1.3 40-50 mg/kg/day**								
Zhang 2020	0.0712	0.027	8	0.0627	0.015	8	0.7%	0.01 [-0.01, 0.03]
Zhao 2013	0.05236	0.00214	10	0.04329	0.0021	10	99.3%	0.01 [0.01, 0.01]
**Subtotal (95% CI)**			**18**			**18**	**100.0%**	**0.01 [0.01, 0.01]**
Heterogeneity: Tau² = 0.00; Chi² = 0.00, df = 1 (P = 0.96); I² = 0%								
Test for overall effect: Z = 9.60 (P < 0.00001)								
**4.1.4 ⩾ 60mg/kg/day**								
Zhang 2020	0.1072	0.049	8	0.0627	0.015	8	30.6%	0.04 [0.01, 0.08]
Zhao 2013	0.05871	0.00124	10	0.04329	0.0021	10	69.4%	0.02 [0.01, 0.02]
**Subtotal (95% CI)**			**18**			**18**	**100.0%**	**0.02 [-0.00, 0.05]**
Heterogeneity: Tau² = 0.00; Chi² = 2.57, df = 1 (P = 0.11); I² = 61%								
Test for overall effect: Z = 1.81 (P = 0.07)								

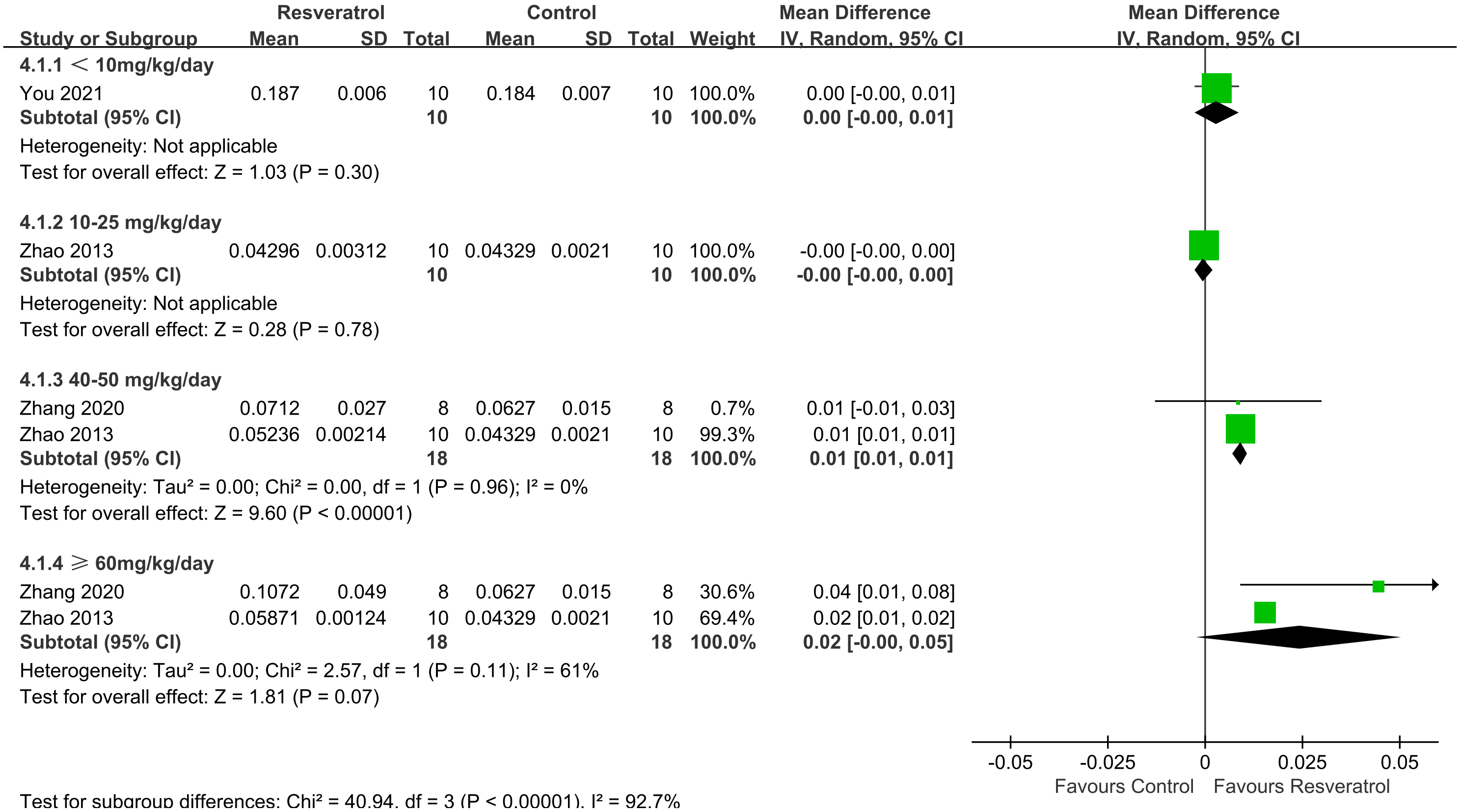

Test for subgroup differences: Chi² = 40.94, df = 3 (P < 0.00001), I² = 92.7%

Supplementary material 2 Forest plot of trabecular spacing.

Study or Subgroup	Resveratrol Mean	Resveratrol SD	Resveratrol Total	Control Mean	Control SD	Control Total	Weight	Mean Difference IV, Random, 95% CI
**6.1.1 ＜10mg/kg/day**								
Feng 2014	0.44	0.04	8	0.49	0.07	8	55.6%	-0.05 [-0.11, 0.01]
You 2021	0.201	0.145	10	0.36	0.02	10	44.4%	-0.16 [-0.25, -0.07]
**Subtotal (95% CI)**			**18**			**18**	**100.0%**	**-0.10 [-0.20, 0.01]**
Heterogeneity: $Tau^2 = 0.00$; $Chi^2 = 4.02$, df = 1 (P = 0.04); $I^2 = 75\%$								
Test for overall effect: Z = 1.82 (P = 0.07)								
**6.1.2 10-25 mg/kg/day**								
Feng 2014	0.28	0.08	8	0.49	0.07	8	100.0%	-0.21 [-0.28, -0.14]
**Subtotal (95% CI)**			**8**			**8**	**100.0%**	**-0.21 [-0.28, -0.14]**
Heterogeneity: Not applicable								
Test for overall effect: Z = 5.59 (P < 0.00001)								
**6.1.3 40-50 mg/kg/day**								
Feng 2014	0.25	0.06	8	0.49	0.07	8	30.5%	-0.24 [-0.30, -0.18]
Feng 2017	0.13	0.02	10	0.24	0.04	10	35.2%	-0.11 [-0.14, -0.08]
Zhang 2020	0.326	0.047	8	0.3691	0.024	8	34.3%	-0.04 [-0.08, -0.01]
**Subtotal (95% CI)**			**26**			**26**	**100.0%**	**-0.13 [-0.21, -0.04]**
Heterogeneity: $Tau^2 = 0.01$; $Chi^2 = 28.21$, df = 2 (P < 0.00001); $I^2 = 93\%$								
Test for overall effect: Z = 2.88 (P = 0.004)								
**6.1.4 ≥60mg/kg/day**								
Zhang 2020	0.1641	0.054	8	0.3691	0.024	8	100.0%	-0.20 [-0.25, -0.16]
**Subtotal (95% CI)**			**8**			**8**	**100.0%**	**-0.20 [-0.25, -0.16]**
Heterogeneity: Not applicable								
Test for overall effect: Z = 9.81 (P < 0.00001)								

Mean Difference IV, Random, 95% CI

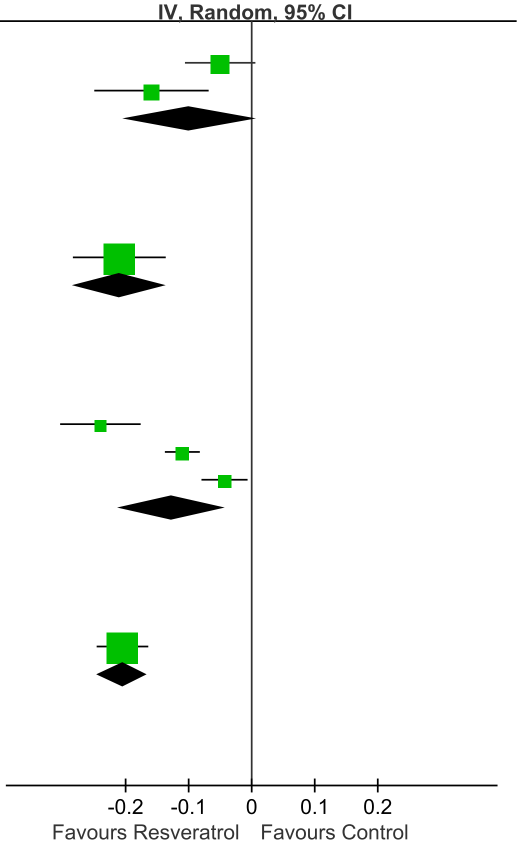

Test for subgroup differences: $Chi^2 = 5.67$, df = 3 (P = 0.13), $I^2 = 47.1\%$

Supplementary material 3 Forest plot of serum phosphorus.

Study or Subgroup	Resveratrol Mean	Resveratrol SD	Resveratrol Total	Control Mean	Control SD	Control Total	Weight	Mean Difference IV, Random, 95% CI
**10.1.1 ＜ 10mg/kg/day**								
Zhang 2012	2.013	0.188	12	2.385	0.249	12	100.0%	-0.37 [-0.55, -0.20]
**Subtotal (95% CI)**			**12**			**12**	**100.0%**	**-0.37 [-0.55, -0.20]**
Heterogeneity: Not applicable								
Test for overall effect: Z = 4.13 (P < 0.0001)								
**10.1.2 10-25 mg/kg/day**								
Li 2016	2.04	0.14	6	2.2	0.19	6	34.7%	-0.16 [-0.35, 0.03]
Wang 2006	1.62	0.22	8	2.73	0.38	4	30.3%	-1.11 [-1.51, -0.71]
Zhang 2012	1.542	0.149	12	2.385	0.249	12	35.0%	-0.84 [-1.01, -0.68]
**Subtotal (95% CI)**			**26**			**22**	**100.0%**	**-0.69 [-1.23, -0.14]**
Heterogeneity: Tau^2 = 0.22; Chi^2 = 35.44, df = 2 (P < 0.00001); I^2 = 94%								
Test for overall effect: Z = 2.46 (P = 0.01)								
**10.1.3 40-50 mg/kg/day**								
Li 2016	1.8	0.17	6	2.2	0.19	6	40.6%	-0.40 [-0.60, -0.20]
Wang 2006	1.96	0.25	7	2.73	0.38	4	14.9%	-0.77 [-1.19, -0.35]
Zhang 2012	1.799	0.216	12	2.385	0.249	12	44.5%	-0.59 [-0.77, -0.40]
**Subtotal (95% CI)**			**25**			**22**	**100.0%**	**-0.54 [-0.71, -0.36]**
Heterogeneity: Tau^2 = 0.01; Chi^2 = 3.18, df = 2 (P = 0.20); I^2 = 37%								
Test for overall effect: Z = 5.99 (P < 0.00001)								
**10.1.4 ⩾ 60mg/kg/day**								
Guo 2015	5.02	0.46	10	5.71	0.46	10	100.0%	-0.69 [-1.09, -0.29]
**Subtotal (95% CI)**			**10**			**10**	**100.0%**	**-0.69 [-1.09, -0.29]**
Heterogeneity: Not applicable								
Test for overall effect: Z = 3.35 (P = 0.0008)								

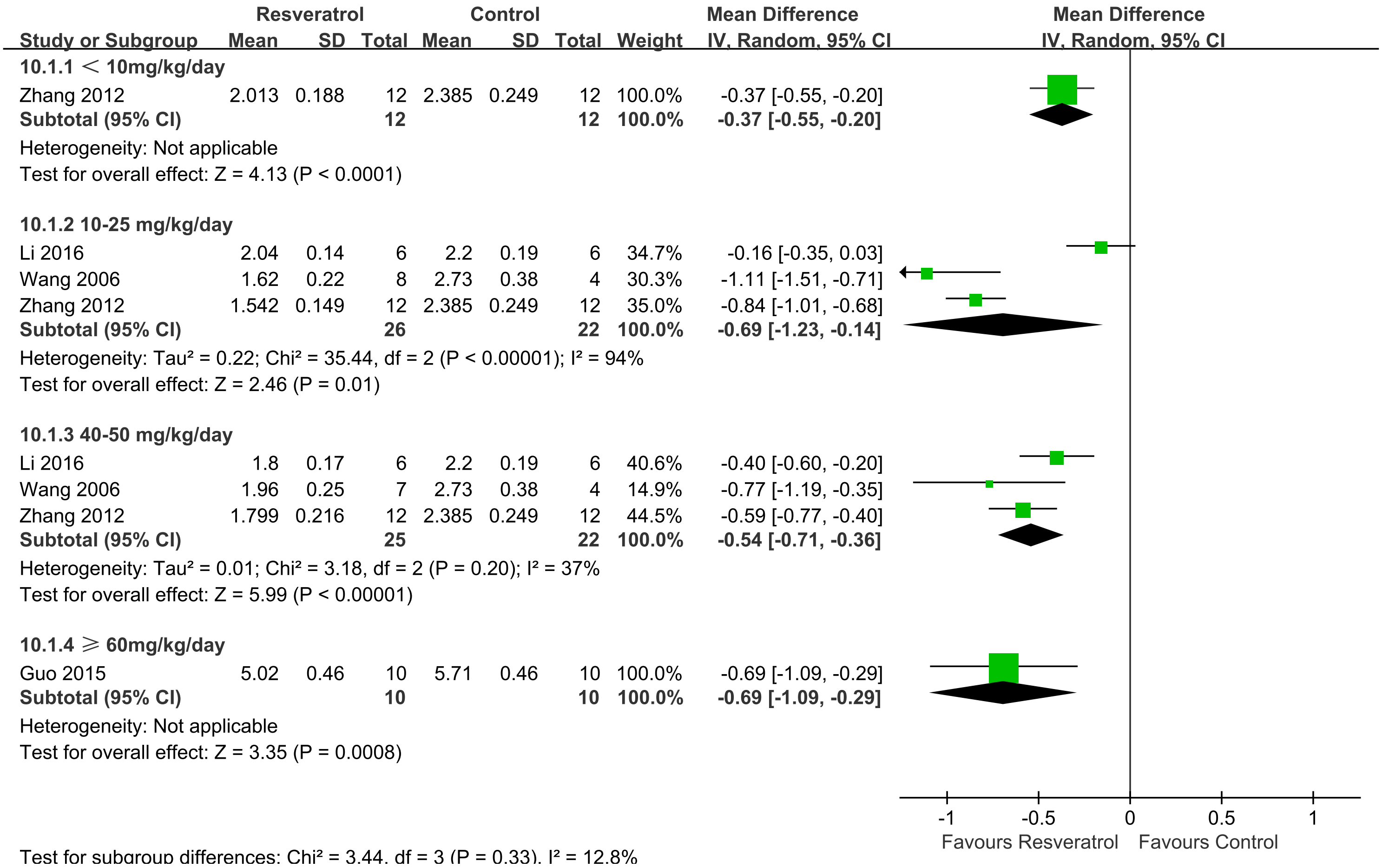

Test for subgroup differences: Chi^2 = 3.44, df = 3 (P = 0.33), I^2 = 12.8%

Supplementary material 4 Forest plot of serum osteocalcin.

	Resveratrol			Control				Mean Difference
**Study or Subgroup**	**Mean**	**SD**	**Total**	**Mean**	**SD**	**Total**	**Weight**	**IV, Random, 95% CI**
**8.1.1 ＜10mg/kg/day**								
Feng 2014	45.5	10.8	8	49.7	9.1	8	49.9%	-4.20 [-13.99, 5.59]
You 2021	221.09	9.94	10	150.5	7.31	10	50.1%	70.59 [62.94, 78.24]
**Subtotal (95% CI)**			**18**			**18**	**100.0%**	**33.26 [-40.03, 106.55]**
Heterogeneity: Tau² = 2776.69; Chi² = 139.30, df = 1 (P < 0.00001); I² = 99%								
Test for overall effect: Z = 0.89 (P = 0.37)								
**8.1.2 10-25 mg/kg/day**								
Feng 2014	40.5	16.4	8	49.7	9.1	8	100.0%	-9.20 [-22.20, 3.80]
**Subtotal (95% CI)**			**8**			**8**	**100.0%**	**-9.20 [-22.20, 3.80]**
Heterogeneity: Not applicable								
Test for overall effect: Z = 1.39 (P = 0.17)								
**8.1.3 40-50 mg/kg/day**								
Feng 2014	37.1	10.9	8	49.7	9.1	8	46.0%	-12.60 [-22.44, -2.76]
Zhang 2022	20.96	2.03	10	16.08	1.83	10	54.0%	4.88 [3.19, 6.57]
**Subtotal (95% CI)**			**18**			**18**	**100.0%**	**-3.16 [-20.24, 13.91]**
Heterogeneity: Tau² = 139.80; Chi² = 11.77, df = 1 (P = 0.0006); I² = 92%								
Test for overall effect: Z = 0.36 (P = 0.72)								
**8.1.4 ⩾60mg/kg/day**								
Guo 2015	2.52	0.47	10	1.71	0.42	10	100.0%	0.81 [0.42, 1.20]
**Subtotal (95% CI)**			**10**			**10**	**100.0%**	**0.81 [0.42, 1.20]**
Heterogeneity: Not applicable								
Test for overall effect: Z = 4.06 (P < 0.0001)								

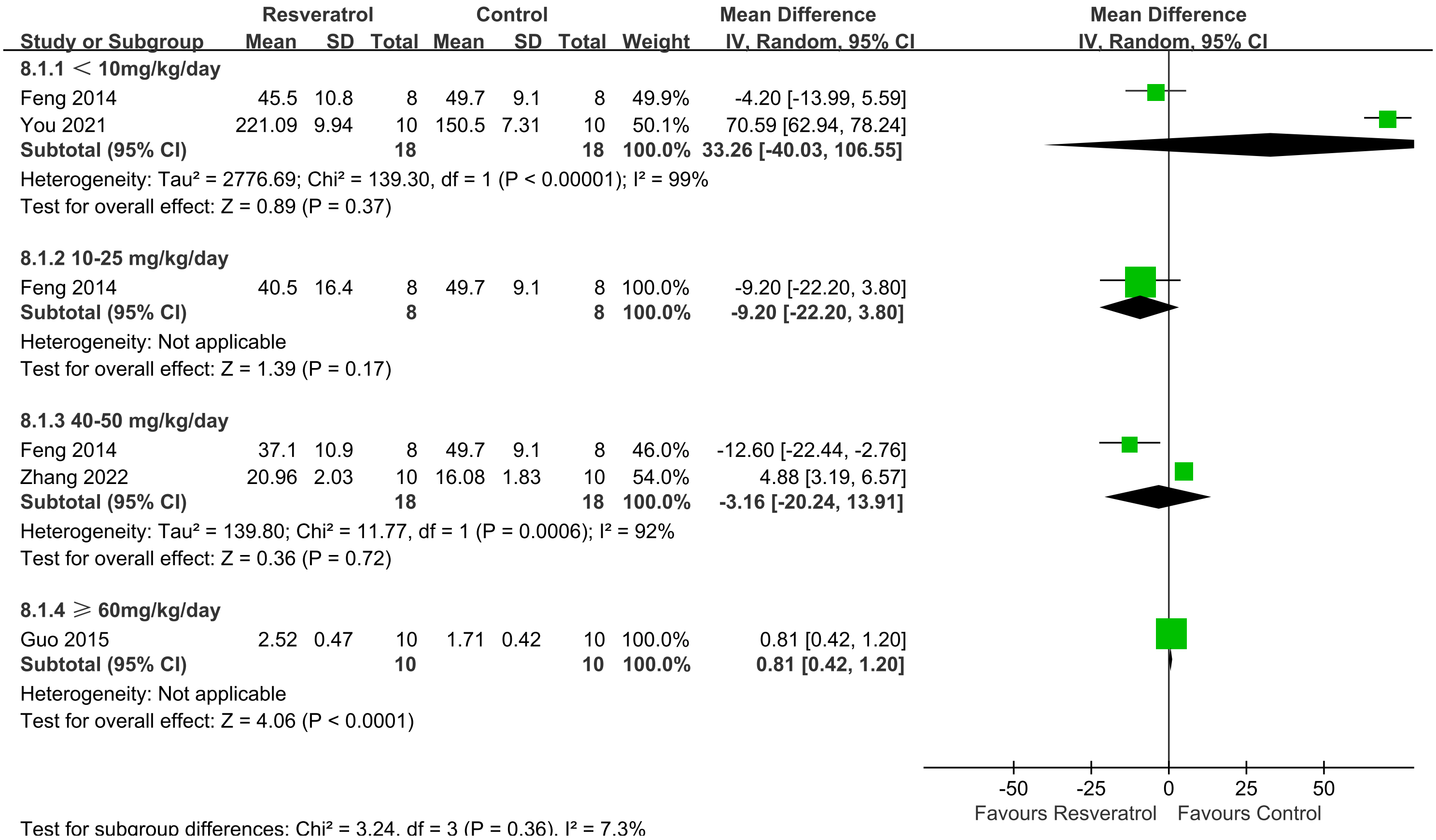

Test for subgroup differences: Chi² = 3.24, df = 3 (P = 0.36), I² = 7.3%

Supplementary material 5 Funnel plot for all outcomes.

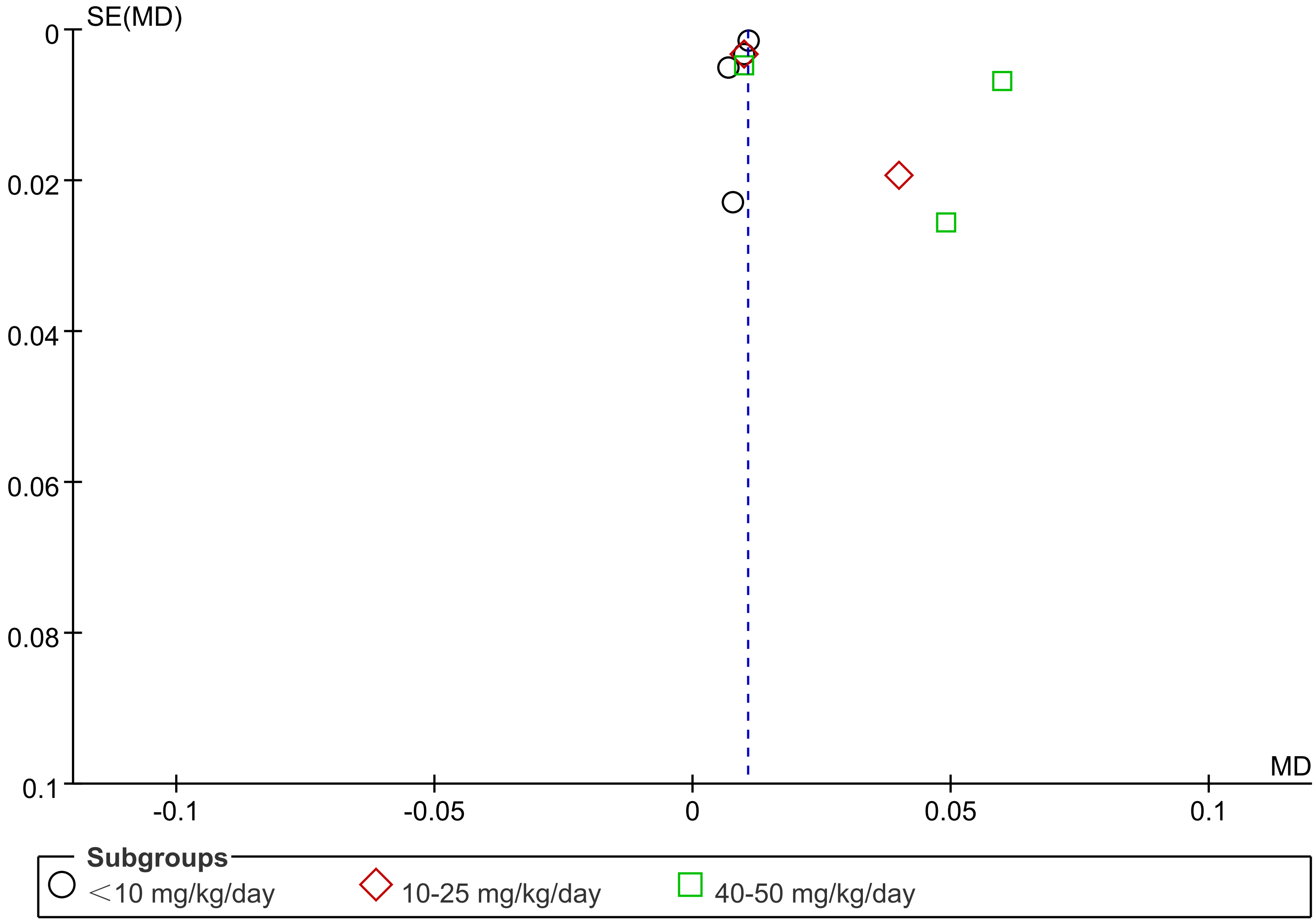

Figure S1 Funnel plot for BMD of the total body.

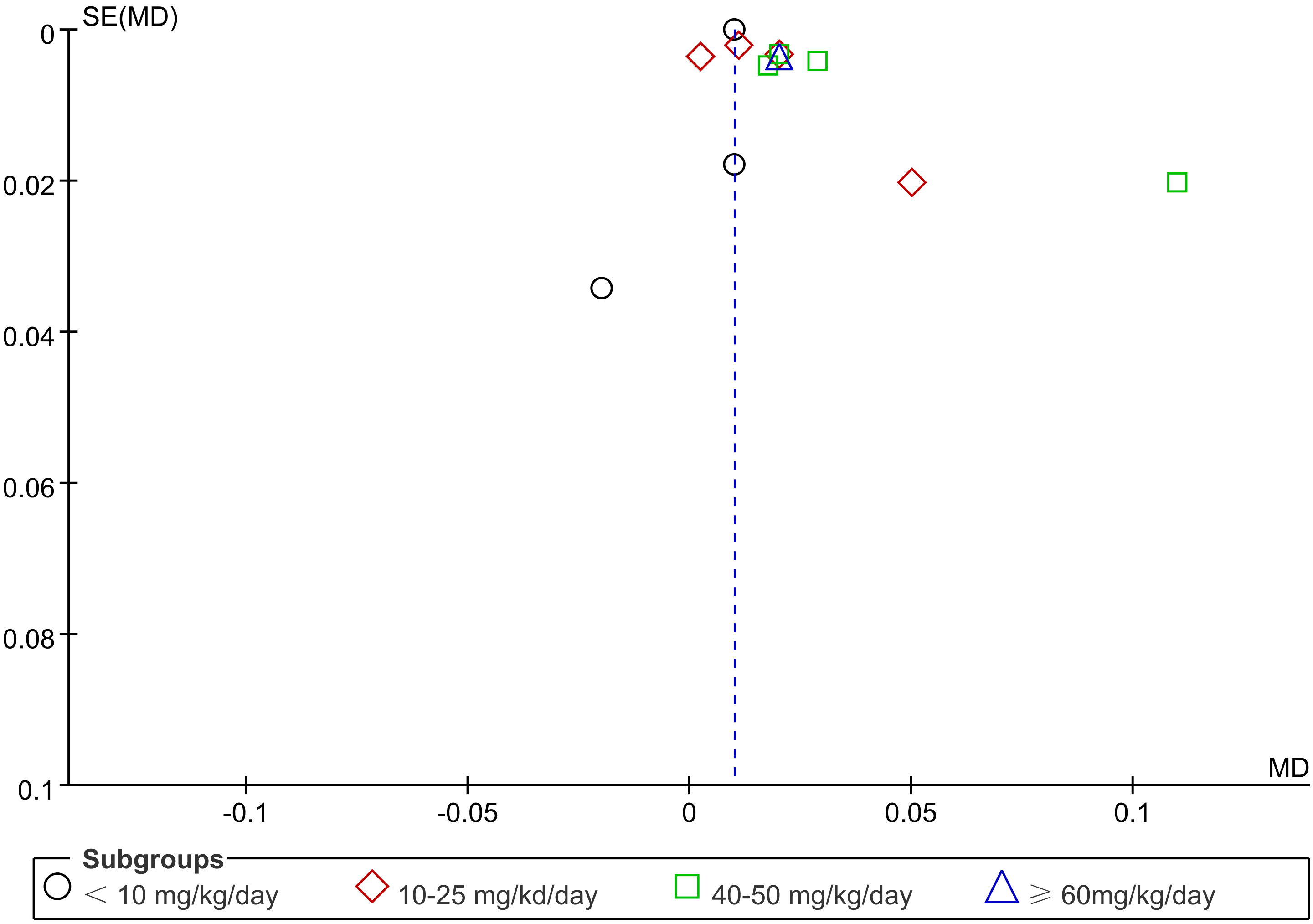

Figure S2 Funnel plot for BMD of the femur.

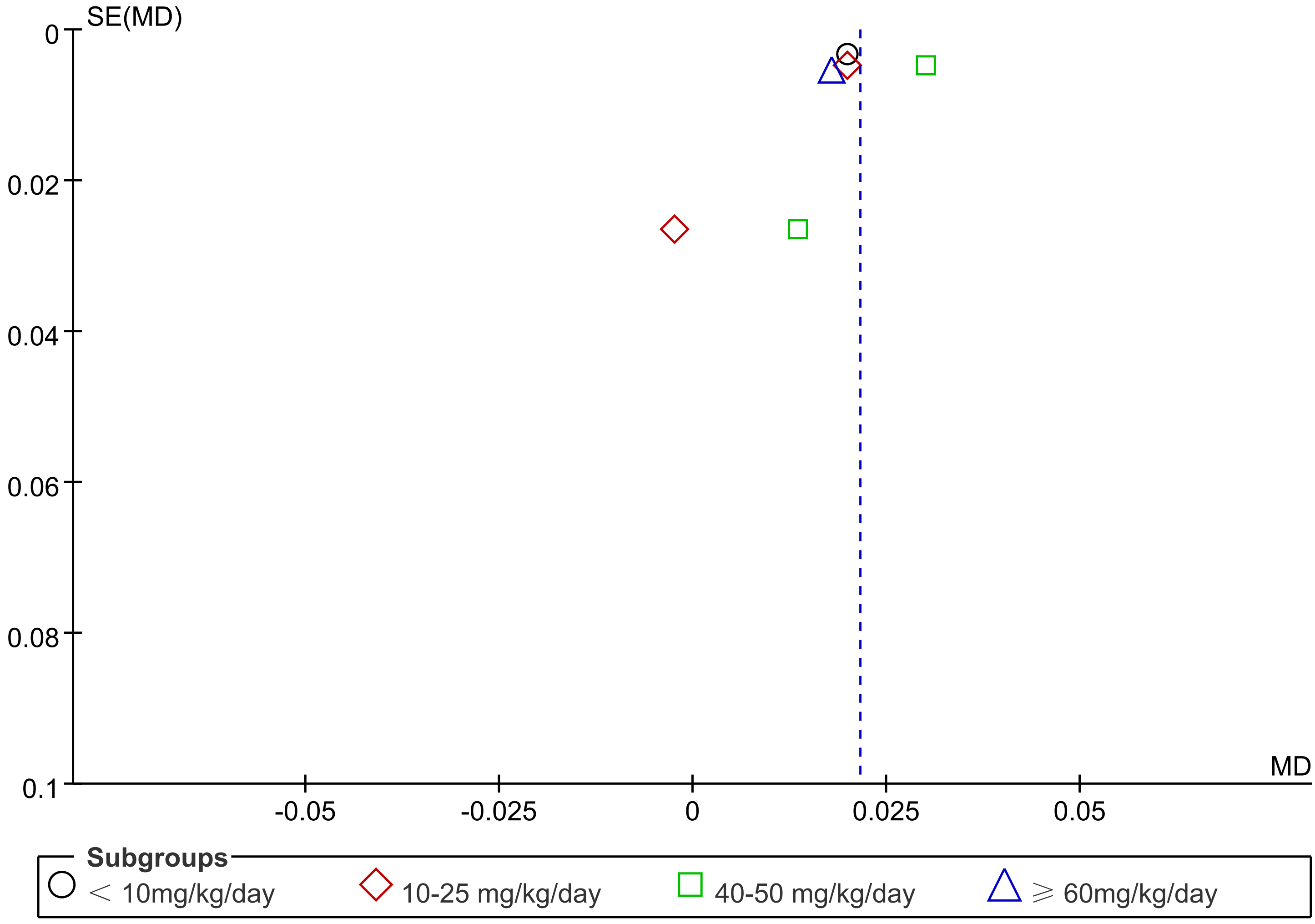

Figure S3 Funnel plot for BMD of the lumbar vertebrae.

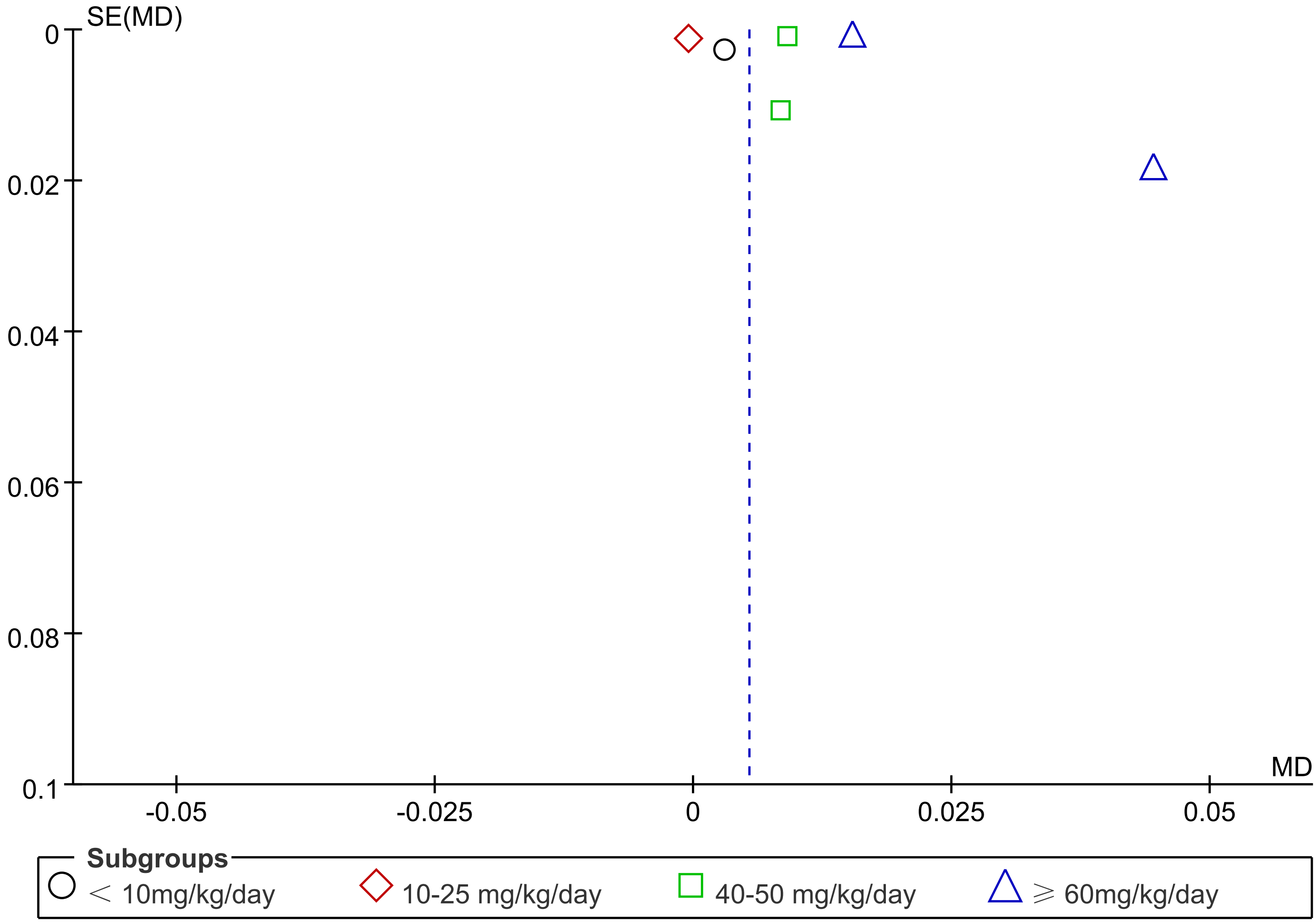

Figure S4 Funnel plot for Trabecular Thickness.

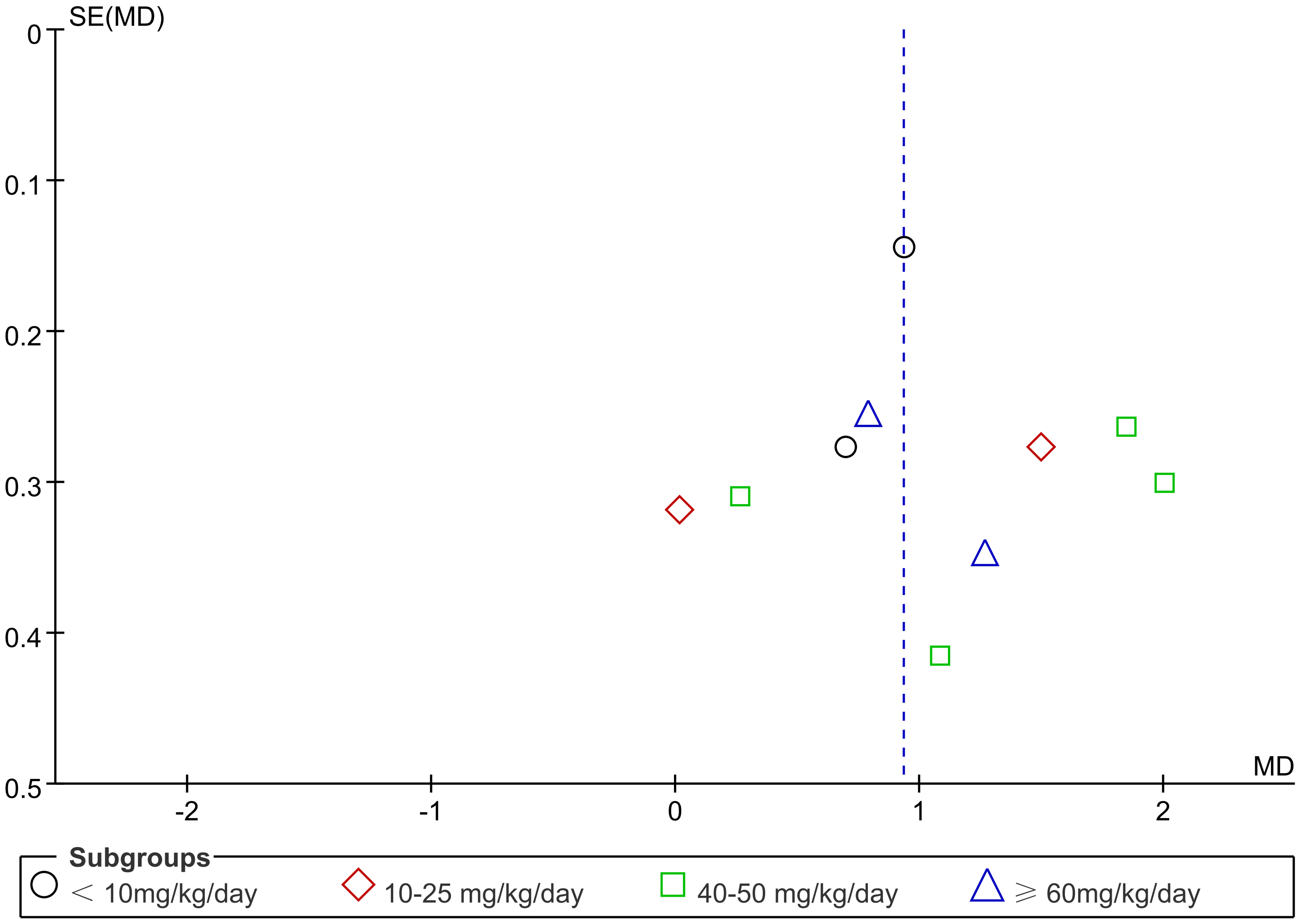

Figure S5 Funnel plot for trabecular number.

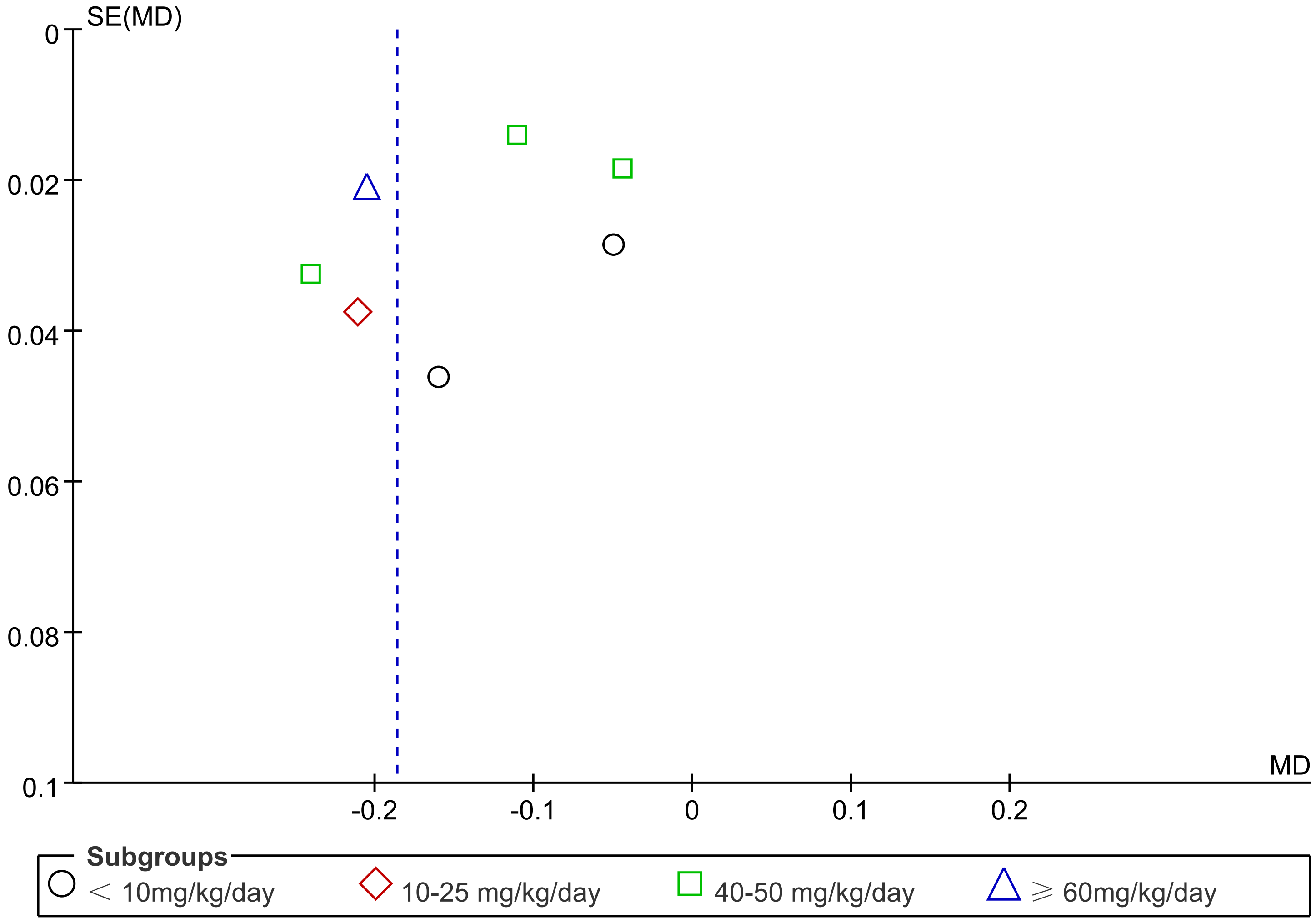

Figure S6 Funnel plot for trabecular spacing.

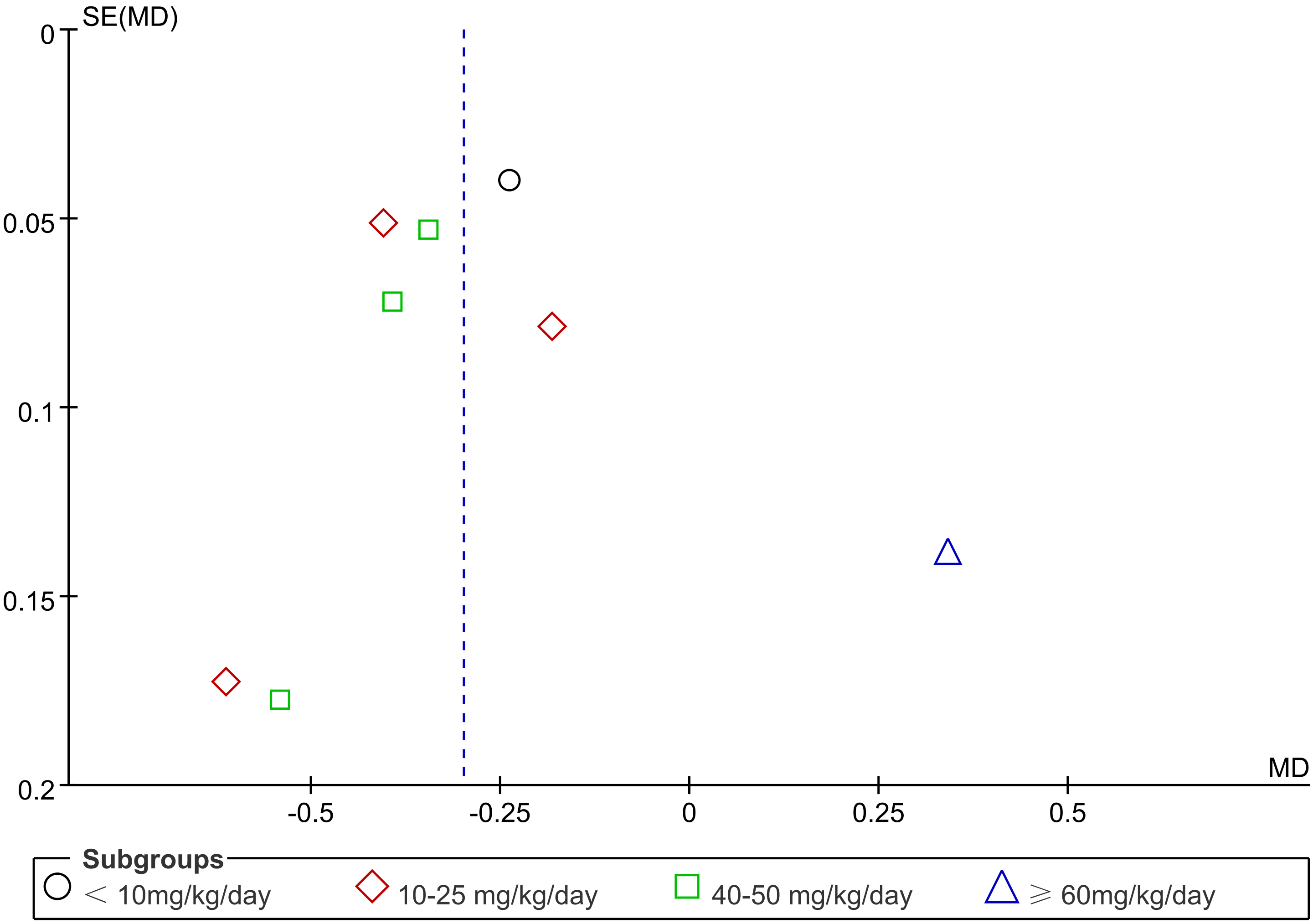

Figure S7 Funnel plot for serum calcium.

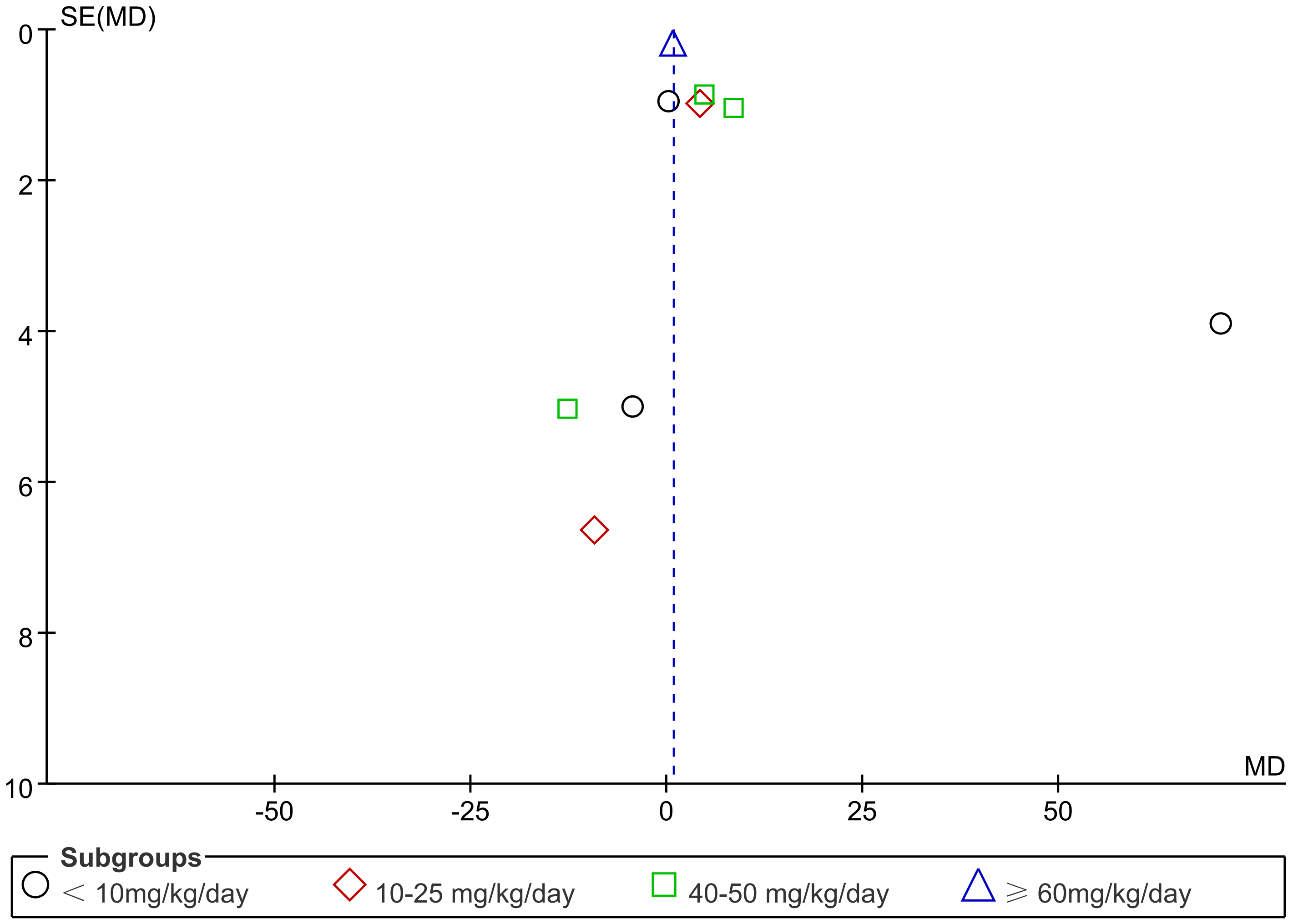

Figure S8 Funnel plot for serum osteocalcin.

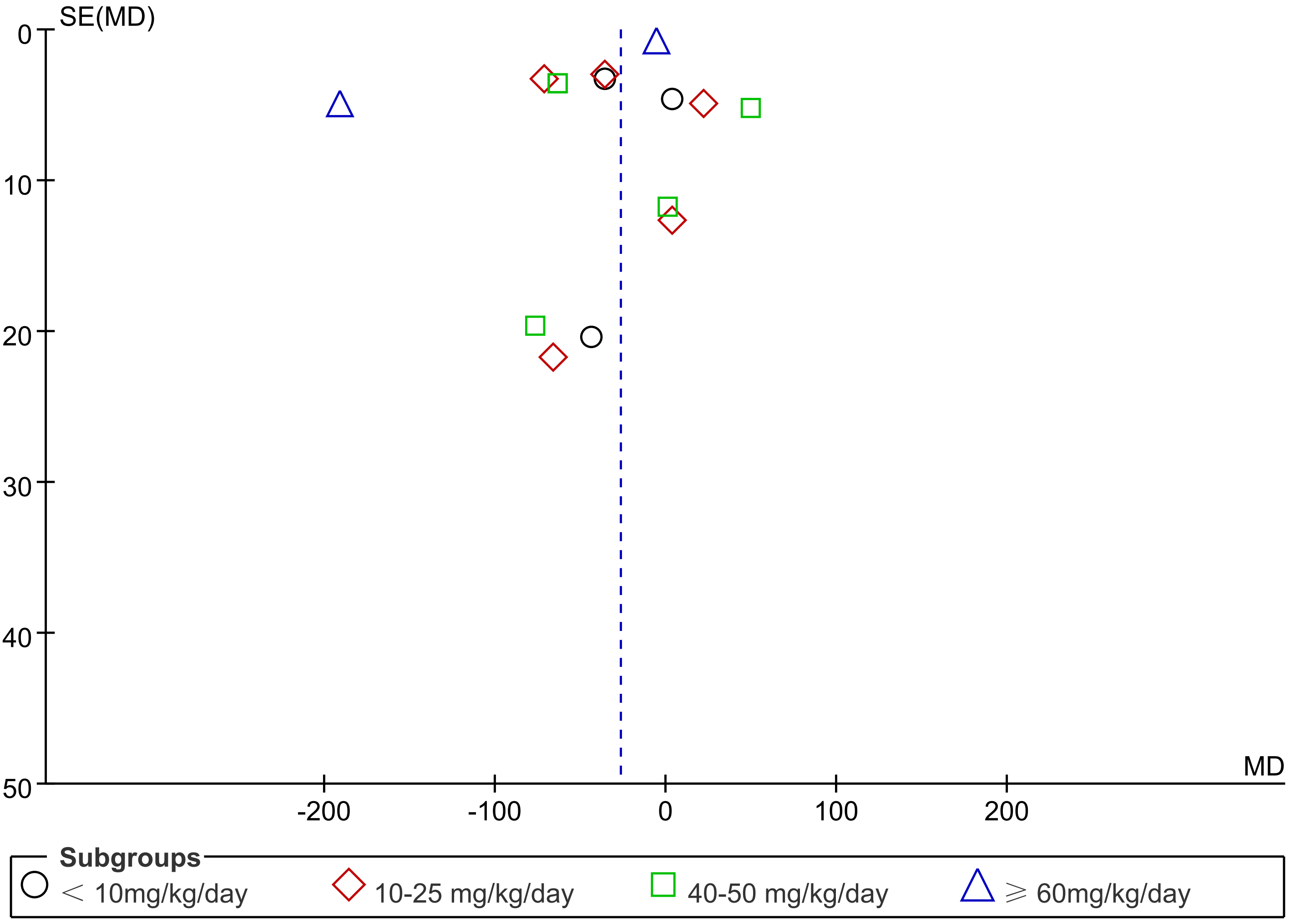

Figure S9 Funnel plot for serum ALP.

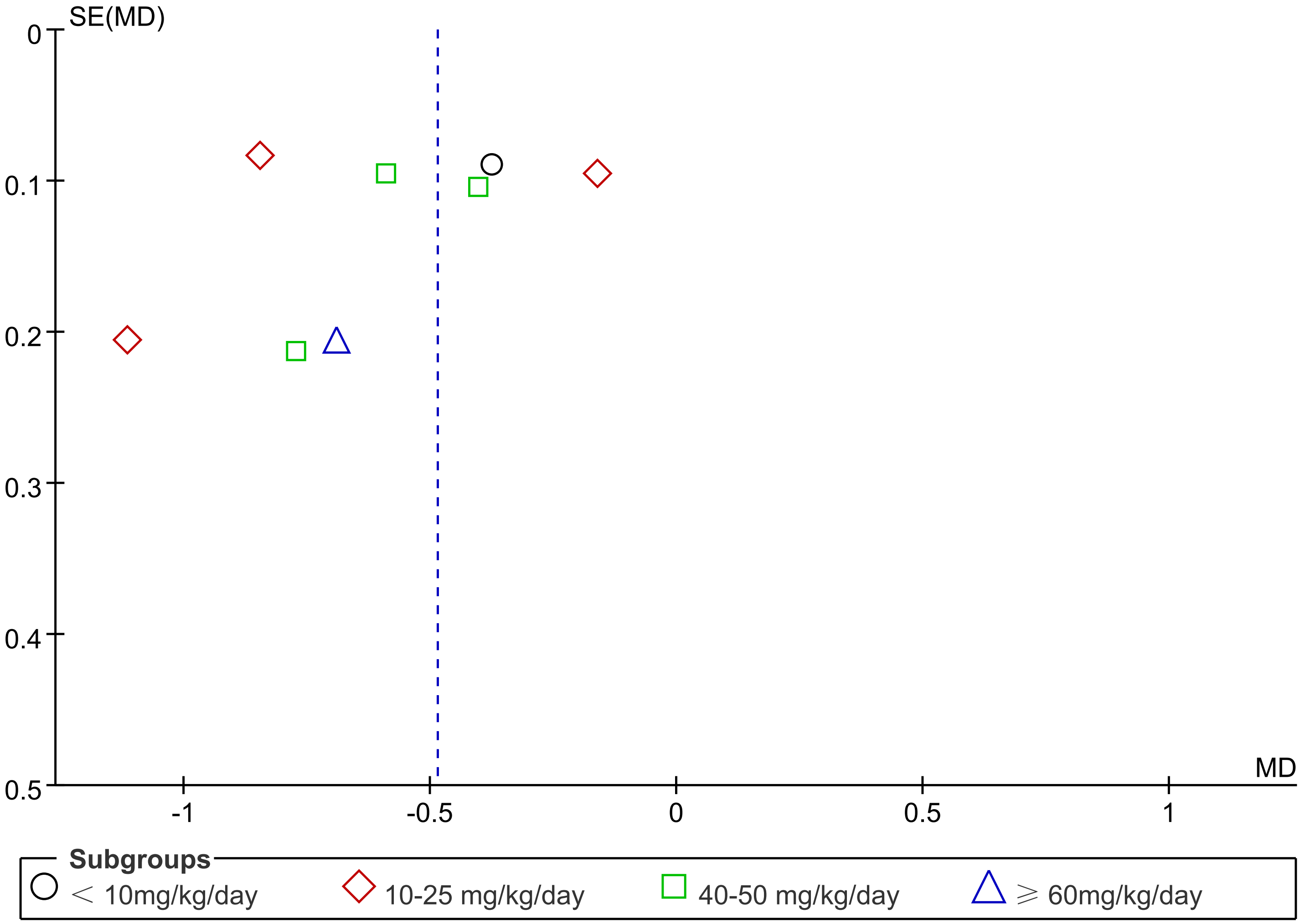

Figure S10 Funnel plot for serum phosphorus.
